# Lactate Attenuates Synaptic Transmission and Affects Brain Rhythms Featuring High Energy Expenditure

**DOI:** 10.1016/j.isci.2020.101316

**Published:** 2020-06-27

**Authors:** Jan-Oliver Hollnagel, Tiziana Cesetti, Justus Schneider, Alina Vazetdinova, Fliza Valiullina-Rakhmatullina, Andrea Lewen, Andrei Rozov, Oliver Kann

**Affiliations:** 1 Institute of Physiology and Pathophysiology, University of Heidelberg, Im Neuenheimer Feld 326, 69120 Heidelberg, Germany; 2 OpenLab of Neurobiology, Kazan Federal University, 420008 Kazan, Russia; 3 Interdisciplinary Center for Neurosciences, University of Heidelberg, 69120 Heidelberg, Germany

**Keywords:** Biochemistry, Neuroscience, Cell Biology

## Abstract

Lactate shuttled from blood, astrocytes, and/or oligodendrocytes may serve as the major glucose alternative in brain energy metabolism. However, its effectiveness in fueling neuronal information processing underlying complex cortex functions like perception and memory is unclear. We show that sole lactate disturbs electrical gamma and theta-gamma oscillations in hippocampal networks by either attenuation or neural bursts. Bursting is suppressed by elevating the glucose fraction in substrate supply. By contrast, lactate does not affect electrical sharp wave-ripple activity featuring lower energy use. Lactate increases the oxygen consumption during the network states, reflecting enhanced oxidative ATP synthesis in mitochondria. Finally, lactate attenuates synaptic transmission in excitatory pyramidal cells and fast-spiking, inhibitory interneurons by reduced neurotransmitter release from presynaptic terminals, whereas action potential generation in the axon is regular. In conclusion, sole lactate is less effective and potentially harmful during gamma-band rhythms by omitting obligatory ATP delivery through fast glycolysis at the synapse.

## Highlights

• Lactate fuels network oscillations featuring low energy expenditure
• Lactate can disturb the neuronal excitation-inhibition balance
• Lactate attenuates neurotransmission at glutamatergic and GABAergic synapses
• Lactate increases oxygen consumption, whereas neural activity can even decrease

## Graphical Abstract

**Figure:**
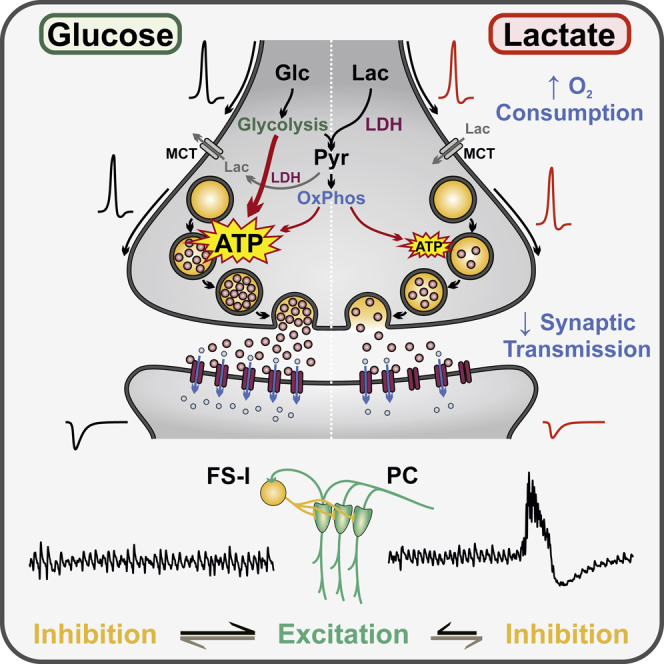

## Introduction

Lactate is a three-carbon, electron-rich metabolite that can be produced and released by various cell types of the body (Brooks, 2018). In utilizing cells, lactate is linked to oxidative ATP synthesis in mitochondria, which requires conversion back to pyruvate through the redox enzyme lactate dehydrogenase (LDH), the tricarboxylic acid cycle, and molecular oxygen serving as the final electron acceptor at the respiratory chain (Brooks, 2018; Dienel, 2019).

Neurons are generally capable of lactate uptake and utilization in mitochondria (Magistretti and Allaman, 2015; Dienel, 2019). Lactate can be released from glial cells, such as astrocytes and oligodendrocytes (Pellerin and Magistretti, 1994; Gandhi et al., 2009; Saab et al., 2016), and it can enter the brain parenchyma from the blood when physical activity increases plasma lactate to as high as 20 mM (Rasmussen et al., 2010; Dienel, 2019). High lactate levels also occur under pathological conditions, such as lactic acidosis, brain ischemia, and traumatic injury (Kraut and Madias, 2014; Glenn et al., 2015; Dienel, 2019). The shuttling of lactate between brain cells depends on various monocarboxylic acid transporters (MCTs) and follows the local concentration gradient (Barros, 2013; Mächler et al., 2016).

Lactate has been reported to support neural survival, evoked neuronal population responses, and synaptic plasticity in a variety of experimental models (Schurr et al., 1988; Izumi et al., 1997; Bouzier-Sore et al., 2003; Suzuki et al., 2011; Wyss et al., 2011; Mächler et al., 2016). In these studies, immature dissociated neuronal cultures, artificial electrical stimulation, excessive lactate concentrations, and/or anesthesia were used. These potential limitations have significantly contributed to the long-lasting and lively controversy about the role of lactate in neuronal energy metabolism (Magistretti and Pellerin, 1999; Chih et al., 2001; Hyder et al., 2006; Harris et al., 2012; Barros, 2013; Magistretti and Allaman, 2015, 2018; Yellen, 2018; Dienel, 2019).

A major issue is that lactate metabolism has been rarely linked to physiological neuronal activity that underlies information processing in the cortex. For example, the role of lactate utilization during different cortical network rhythms, which naturally occur during cognition and behavior *in vivo*, is widely unknown (Colgin, 2016; Dienel, 2019; Scheeringa and Fries, 2019). Similarly, lactate utilization in excitatory neurons and inhibitory interneurons, which can, based on their specific functions, substantially differ in electrophysiological and bioenergetic properties, has been barely explored (Buzsáki et al., 2007; Kann, 2016). Related to that, the necessity of fast glycolytic ATP supply during neuronal signaling, in particular the presynaptic vesicle filling with neurotransmitters, is not well established (Ikemoto et al., 2003; Hall et al., 2012; Ashrafi and Ryan, 2017; Lucas et al., 2018).

We addressed these fundamental issues by exploring lactate utilization at the neuronal network and single cell level in slice preparations of the hippocampus. We examined *ex vivo* slices from young adult rats and slice cultures from rat pups in interface (extracellular local field potential and oxygen recordings) or submerged (intracellular patch-clamp recordings) conditions (Discussion, Limitations of the Study, and Transparent Methods: Recording solution and drugs). We focused on two fast network rhythms: (1) gamma oscillations (30–70 Hz) that emerge in many cortical areas in awake mammals during perception, locomotion, and memory formation (Melloni et al., 2007; van Vugt et al., 2010; Colgin, 2016), and (2) sharp wave-ripples (>180 Hz) that arise during waking immobility and slow-wave sleep and likely assist in memory consolidation (Buzsáki, 2015; Ramirez-Villegas et al., 2015). Both rhythms rely on precise mutual synaptic transmission between excitatory pyramidal cells and GABAergic interneurons (Hájos and Paulsen, 2009; Colgin, 2016).

In essence, we demonstrate that sole lactate is less effective than glucose in fueling gamma and theta-gamma oscillations and identify attenuated synaptic transmission because of reduced neurotransmitter release as the main mechanistic cause.

## Results

### Energetic Boundary Conditions during Gamma Oscillations in *Ex Vivo* Slices

In the normal brain, the concentrations of glucose and lactate in the extracellular space are approximately 2 and 3 mM, respectively (Zilberter et al., 2010). In brain slice preparations, glucose and lactate at 2–3 mM have been shown to maintain energy metabolism and thus synaptic function under special experimental conditions (Schurr et al., 1988; Ivanov et al., 2014; Díaz-García et al., 2017). We first tested whether fast neuronal network oscillations in the gamma band (30–70 Hz) tolerate energy substrate concentrations closer to the physiological range in *ex vivo* slices of the hippocampus. Notably, gamma oscillations associate with high energy expenditure (Niessing et al., 2005; Kann et al., 2011; Schneider et al., 2019).

**Figure 1:**
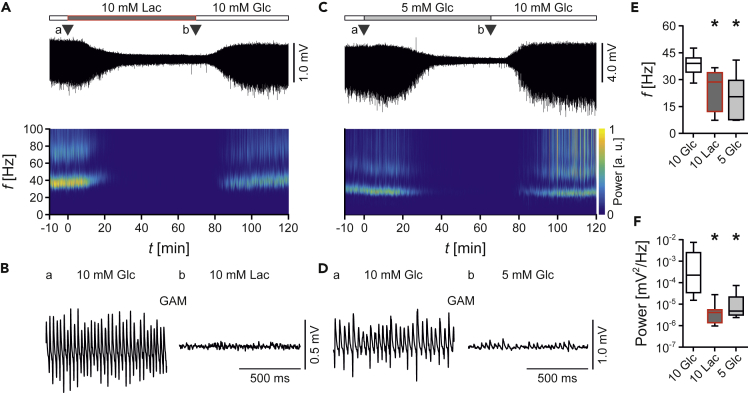
Energetic Boundary Conditions during Gamma Oscillations Local field potentials were recorded in stratum pyramidale of CA3 in *ex vivo* slices. Gamma oscillations (GAM) were induced by bath application of acetylcholine (10 μM) and physostigmine (2 μM). (A) Sample trace of gamma oscillations fueled by glucose (Glc) or lactate (Lac) and corresponding wavelet transformation showing power of frequency domains (*f*) over time (*t*). Heat-scale colors encode for power (Power) in arbitrary units (a.u.). Triangles (a and b) indicate recording segments shown at higher temporal resolution in (B). (B) Sample traces of gamma oscillations in (a) glucose (10 mM) or (b) lactate (10 mM). (C and D) (C) Same as in (A) and (D) same as in (B) in (a) standard glucose (10 mM) or (b) low glucose (5 mM). (E and F) Gamma oscillations were analyzed for frequency and power. *n/N* (slices/animals): 10 Glc, 17/5; 10 Lac, 9/3; 5 Glc, 8/3. (E) Peak frequency (*f*). Each ∗p < 0.05 versus 10 Glc, Kruskal-Wallis with Dunn's multiple comparisons test. (F) Peak of power spectral density (Power). Each ∗p < 0.05 versus 10 Glc, Kruskal-Wallis with Dunn's multiple comparisons test. Data are given as median ± interquartile range (IQR = 75% percentile - 25% percentile), error bars indicate minimal and maximal values.

Highly synchronized gamma oscillations were present in stratum pyramidale of the CA3 region under control conditions with standard glucose (10 mM). These oscillations share many properties with gamma oscillations *in vivo* (Hájos and Paulsen, 2009; Gulyás et al., 2010; Kann et al., 2011). By contrast, 5 mM glucose or 10 mM lactate resulted in suppression of gamma oscillations, which was widely reversible (Figures 1A–1D). Specifically, there were clear decreases in frequency and power of the oscillations (Figures 1E and 1F). These strong effects did not permit reliable analysis of synchronization and inner coherence of the oscillations (Transparent Methods: Data analysis). However, they likely reflect the large fall in the glucose concentration from the slice surface (10 mM) to the slice core (about 3 mM) in *ex vivo* slices (Lourenço et al., 2019).

These data show that gamma oscillations in *ex vivo* slices require a large substrate concentration gradient from the ambient recording solution to the slice core because of high energy expenditure and longer diffusion distances inherent to slice preparations (Kann and Kovács, 2007) (Discussion, Limitations of the Study, and Transparent Methods: Recording solution and drugs).

### Lactate Evokes Neural Bursts during Highly Synchronized Gamma Oscillations

Previous studies suggested that lactate is an alternative — and even preferred — energy substrate of neurons to maintain survival and synaptic function (Schurr et al., 1988; Izumi et al., 1997; Bouzier-Sore et al., 2003; Wyss et al., 2011). We next tested whether highly synchronized gamma oscillations can be fueled with lactate at a concentration that mimics sufficient substrate supply in our experimental conditions. By approximation, we used the 2-fold concentration of lactate (two lactate molecules can be derived from one glucose molecule), emphasizing that 20 mM lactate and 10 mM glucose are not isocaloric (Limitations of the Study).

**Figure 2:**
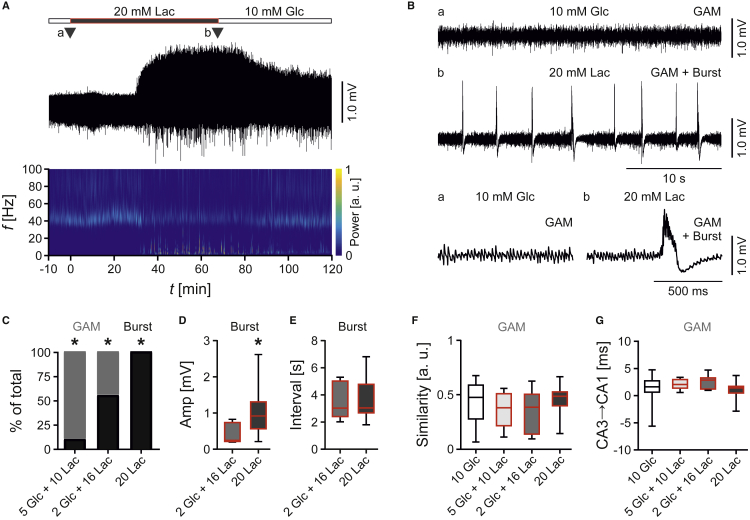
Lactate-Evoked Neural Bursts Local field potentials were recorded in stratum pyramidale of CA3 in *ex vivo* slices. Gamma oscillations (GAM) were induced by bath application of acetylcholine (10 μM) and physostigmine (2 μM). (A) Sample trace of gamma oscillations fueled by glucose (Glc, 10 mM) or lactate (Lac, 20 mM) and corresponding wavelet transformation showing power of frequency domains (*f*) over time (*t*). Heat-scale colors encode for power (Power) in arbitrary units (a.u.). Triangles (a and b) indicate recording segments shown at higher temporal resolution in (B). (B) Sample traces of gamma oscillations (a) without and (b) with recurrent neural bursts. (C–G) Network activities were analyzed for different parameters. *n/N* (slices/animals): 10 Glc, 34/11; 5 Glc + 10 Lac, 9/5; 2 Glc + 16 Lac, 9/5; 20 Lac, 17/8. (C) The incidence of bursts (black) increases with the fraction of lactate in the recording solutions. Each ∗p < 0.05 in all tested combinations, chi-square test. The onset of bursts was at 35 ± 8 min (mean ± SEM) (2 Glc + 16 Lac) and 37 ± 4 min (20 Lac). p = 0.82, unpaired t test. (D) Amplitude of bursts (Amp). ∗p < 0.05, unpaired t test. (E) Interval of bursts (Interval), Mann-Whitney test. (F) Similarity of gamma oscillations in CA3 and CA1 (Similarity), Kruskall-Wallis with Dunn's multiple comparisons test. (G) Time lag of gamma oscillations between CA3 and CA1 (CA3→CA1), indicating oscillation propagation. Kruskall-Wallis with Dunn's multiple comparisons test. Data are given as median ± interquartile range (IQR = 75% percentile - 25% percentile), error bars indicate minimal and maximal values. See also Figures S1 and S2.

Strikingly, lactate (20 mM) evoked recurrent neural bursts with an incidence of about 0.3/s that were superimposed onto gamma oscillations (Figures 2A and 2B). The amplitude of these bursts indicates moderate hyperexcitability rather than epileptiform discharges that can be evoked in these slices (Liotta et al., 2011). Indeed, moderate pharmacological disinhibition through GABA_A_-receptors also associated with neural bursting (Figure S1). When increasing the fraction of glucose from 0 to 2 or 5 mM in energy substrate supply, the lactate effect was reversible for the number of slices that expressed neural bursts and for the burst amplitudes; the burst intervals were unchanged (Figures 2C–2E). Further analysis did not reveal any signs of desynchronization or altered spatial propagation of gamma oscillations prior to the onset of the first neural burst (Figures 2F, 2G, and S2).

These data show that sole lactate disturbs highly synchronized gamma oscillations, likely by an excitation-inhibition imbalance in the local neuronal network. However, gamma oscillations can be fueled by supplemental lactate when a low amount of glucose is available.

### Lactate Attenuates Less Synchronized Gamma Oscillations

Gamma oscillations can also be reliably induced in postnatal slice cultures of the hippocampus that permit experimental conditions with improved supply of oxygen and energy substrates (Kann et al., 2011; Huchzermeyer et al., 2013). We next tested how lactate affects gamma oscillations in slice cultures at ambient normoxia (20% oxygen fraction) as well as in the presence of theta oscillations that often occur simultaneously *in vivo* (Hájos and Paulsen, 2009; Colgin, 2016).

**Figure 3:**
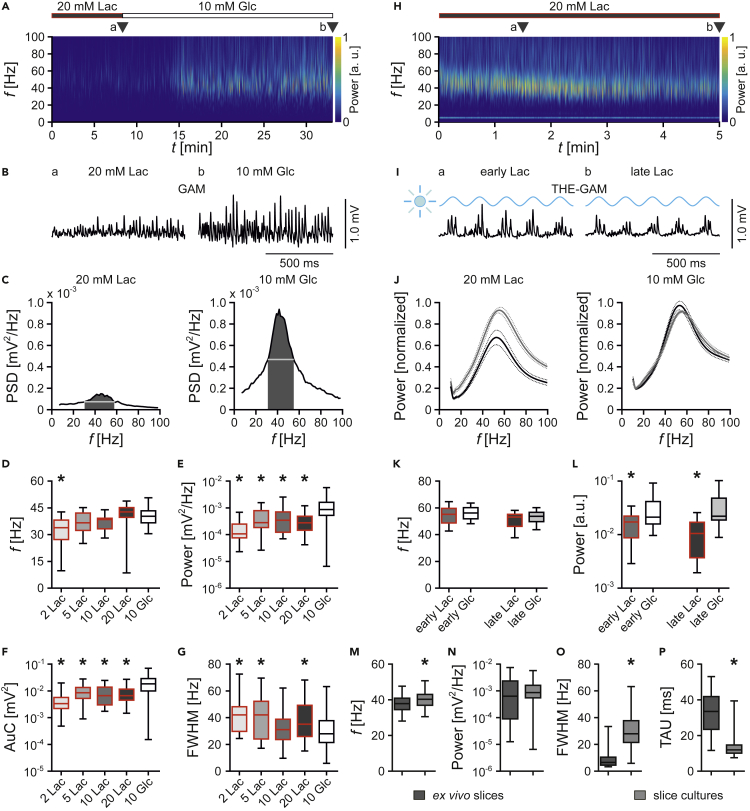
Lactate-Evoked Attenuation of Gamma and Theta-Gamma Oscillations Local field potentials were recorded in stratum pyramidale of CA3 in slice cultures. In (A)–(G), gamma oscillations (GAM) were induced by bath application of acetylcholine (2 μM) and physostigmine (0.4 μM). In (H)–(L), theta-gamma oscillations (THE-GAM) were optogenetically induced. (A) Wavelet transformation showing power of frequency domains (*f*) over time (*t*) of a sample trace of gamma oscillations fueled by lactate (Lac, 20 mM) or glucose (Glc, 10 mM). Heat-scale colors encode for power (Power) in arbitrary units (a.u.). Triangles (a and b) indicate recording segments shown at higher temporal resolution in (B). (B) Sample traces of gamma oscillations in (a) lactate or (b) glucose. (C) Power spectra indicating the power spectral density (PSD) of conditions (last 5 min) in (A). (D–G) Gamma oscillations recorded at various lactate concentrations (2, 5, 10, and 20 mM) were analyzed for different parameters. *n/N* (slices/preparations): 2 Lac, 16/3; 5 Lac, 19/3; 10 Lac, 18/3; 20 Lac, 38/3; 10 Glc, 91/12. (D) Peak frequency (*f*). (E) Peak of power spectral density (Power). (F) Area under the curve (AuC, dark gray areas in [C]). (G) Full width at half maximum (FWHM, white horizontal lines in [C]). Each ∗p < 0.05 versus 10 Glc (control), Kruskall-Wallis with Dunn's multiple comparisons test (D–F) and one-way ANOVA with Holm-Šídák's multiple comparisons test (G). (H) Wavelet transformation of optogenetically induced theta-gamma oscillations fueled by lactate (Lac, 20 mM). Triangles (a and b) indicate recording segments shown at higher temporal resolution in (I). (I) Sample traces of theta-gamma oscillations (THE-GAM) in lactate (20 mM) at (a) 1.5 min (early Lac) and (b) 5 min (late Lac) of light stimulation (470 nm, blue sinusoidal curve). (J) Mean of normalized power (Power) derived from wavelet transformation in (H) in lactate (Lac, 20 mM) or glucose (Glc, 10 mM) during early (gray lines) or late (black lines) stages of the recordings (data segments of 1 min). Data are represented by mean (solid lines) ± SEM (dotted lines). (K and L) Theta-gamma oscillations were analyzed for frequency and power. *n/N*: 20 Lac, 15/5; 10 Glc, 19/3. (K) Peak frequency (*f*). One-way ANOVA with Holm-Šídák's multiple comparisons test. (L) Peak of power (Power) in arbitrary units (a.u.). ∗p < 0.05, Kruskall-Wallis with Dunn's multiple comparisons test. (M–P) Comparison of gamma oscillations in glucose (10 mM) in *ex vivo* slices (*n/N*: 48/16) and slice cultures (*n/N*: 91/12). (M) Frequency as in (D). ∗p < 0.05, unpaired t test. (N) Power as in (E). Mann-Whitney test. (O) FWHM as in (G). ∗p < 0.05, Mann-Whitney test. (P) Time constant (TAU) of the decaying exponential fit to the peaks of the autocorrelation. ∗p < 0.05, Mann-Whitney test. Data are given as median ± interquartile range (IQR = 75% percentile - 25% percentile), error bars indicate minimal and maximal values.

Gamma oscillations were induced in lactate (2–20 mM) that was later on replaced by standard glucose (10 mM) serving as control (Figures 3A–3C). Lactate generally attenuated gamma oscillations. Specifically, power (Figure 3E) and synchronization (Figures 3F and 3G) decreased stronger than frequency (Figure 3D). Similarly, theta-gamma oscillations evoked by optogenetic tools (Figures 3H–3J) had a significantly lower power in lactate (20 mM) (Figure 3L). The frequency was unaffected (Figure 3K).

Although gamma oscillations in *ex vivo* slices and in slice cultures share many properties, such as generation in the CA3 region and frequencies at around 40 Hz (Hájos and Paulsen, 2009; Gulyás et al., 2010; Kann et al., 2011), we found that gamma oscillations in slice cultures showed less synchronization and inner coherence (Figures 3M–3P).

These data show that lactate attenuates less synchronized gamma and theta-gamma oscillations.

### Lactate Does Not Affect Sharp Wave-Ripples

We next tested whether the aforementioned disturbances evoked by lactate were specific for gamma oscillations and focused on intermittent sharp wave-ripples that occur *in vivo* and in *ex vivo* slices (Behrens et al., 2005; Ramirez-Villegas et al., 2015).

**Figure 4:**
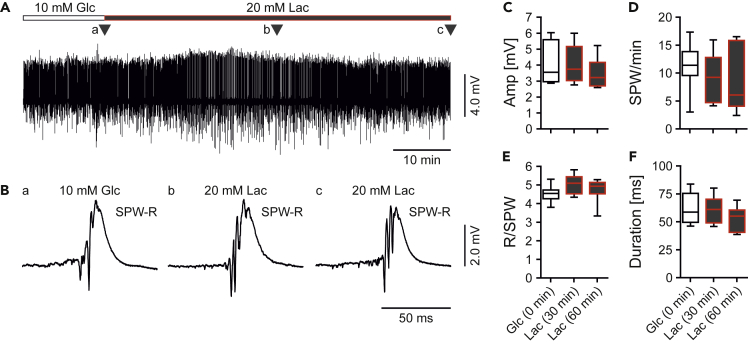
Regular Sharp Wave-Ripples in Lactate Local field potentials were recorded in stratum pyramidale of CA3 in *ex vivo* slices. (A) Sample trace of sharp wave-ripples (SPW-R) fueled by glucose (Glc) or lactate (Lac). Triangles (a–c) indicate recording segments shown at higher temporal resolution in (B). (B) SPW-R events in (a) glucose (10 mM) and after (b) 30 min and (c) 60 min in lactate (20 mM). (C–F) SPW-R were analyzed for different parameters. *n/N* (slices/animals): 8/8. Note that >300 events were analyzed per experimental group. (C) Amplitude of SPW (Amp). Kruskall-Wallis with Dunn's multiple comparisons test. (D) Number of sharp waves (SPW) per min. Kruskall-Wallis with Dunn's multiple comparisons test. (E) Number of ripples per SPW (R/SPW). Kruskall-Wallis with Dunn's multiple comparisons test. (F) Duration of single SPW (Duration). One-way ANOVA with Holm-Šídák's multiple comparisons test. Note that the properties of SPW-R recorded in the presence of lactate are not significantly different from those recorded in glucose. Data are given as median ± interquartile range (IQR = 75% percentile - 25% percentile), error bars indicate minimal and maximal values.

Sharp wave-ripples were stable in standard glucose (10 mM) (Figures 4A and 4B). The sharp waves occurred with an incidence of about 12/min (Figure 4D) and had a superimposed, fast periodic oscillatory component with frequencies of >180 Hz (“ripples”). These properties were similar to sharp wave-ripples in previous reports (Behrens et al., 2005; Hollnagel et al., 2014; Schlingloff et al., 2014). Remarkably, we did not detect any significant differences in the properties of sharp wave-ripples when fueled by sole lactate, even when lactate was present for 1 h (Figures 4C–4F). This persistence of sharp wave-ripples also permitted to quantify adaptations in oxygen metabolism associated with lactate utilization at the same level of network activity (see below).

These data show that lactate specifically disturbs gamma and theta-gamma oscillations.

### Lactate Increases the Oxygen Consumption

The energetic utilization of lactate in neurons requires lactate uptake through MCT-2 and conversion to pyruvate through LDH-1 (Chih et al., 2001; Magistretti and Allaman, 2015). The subsequent oxidative ATP synthesis in mitochondria consumes molecular oxygen (Kann and Kovács, 2007; Dienel, 2019). We next investigated the oxygen metabolism associated with lactate supply. We used O_2_ microsensors to determine local O_2_ concentrations in the tissue (Huchzermeyer et al., 2013; Schneider et al., 2019).

**Figure 5:**
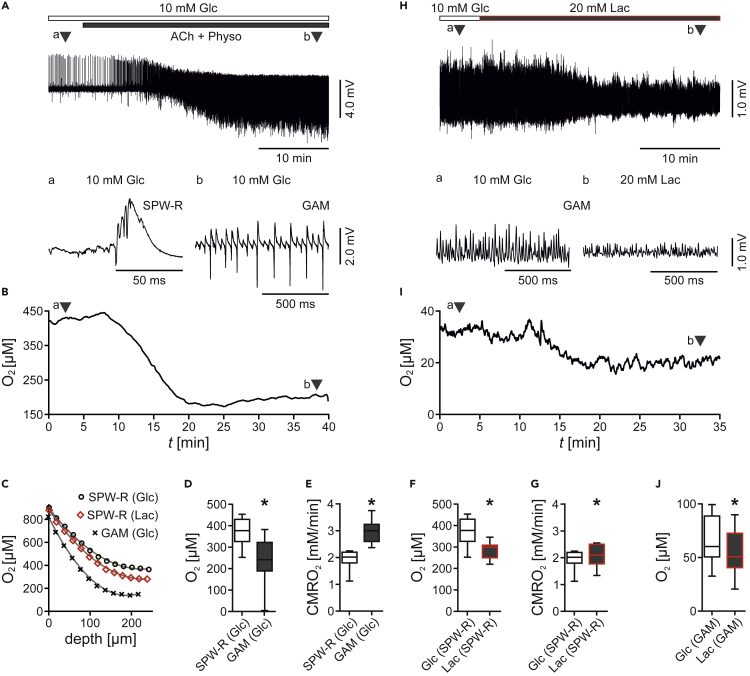
Lactate-Evoked Increase in Oxygen Consumption Local field potentials and tissue O_2_ concentrations were recorded in stratum pyramidale of CA3 in *ex vivo* slices (A–G) and slice cultures (H–J). Gamma oscillations (GAM) were induced by bath application of acetylcholine (ACh, *ex vivo* slices: 10 μM, and slice cultures: 2 μM) and physostigmine (Physo, *ex vivo* slices: 2 μM, and slice cultures: 0.4 μM). (A) Sample trace of sharp wave-ripples (SPW-R) and gamma oscillations fueled by glucose (Glc, 10 mM) in *ex vivo* slices. Triangles (a and b) indicate recording segments shown at higher temporal resolution (bottom). (B) Sample trace of the decrease in O_2_ concentration associated with the switch from (a) SPW-R to (b) GAM (ambient atmosphere with 95% oxygen, O_2_ microsensor placed at 200 μm depth) over time (*t*). (C) Sample oxygen depth profiles recorded during SPW-R or GAM in glucose (Glc, 10 mM) or lactate (Lac, 20 mM). *n/N* (slices/animals): SPW-R (Glc), 8/3; SPW-R (Lac), 8/3; GAM (Glc), 12/5. (D and E) (D) Lowest O_2_ concentration (O_2_) and (E) calculated cerebral metabolic rate of oxygen (CMRO_2_) during SPW-R and gamma oscillations in glucose (10 mM). Each ∗p < 0.05, unpaired t test. (F and G) (F) Lowest O_2_ concentration (O_2_) and (G) calculated CMRO_2_ during SPW-R in glucose (10 mM) and lactate (20 mM). Each ∗p < 0.05, paired t test. (H) Sample trace of gamma oscillations fueled by glucose (Glc, 10 mM) or lactate (Lac, 20 mM) in slice cultures. Triangles (a and b) indicate recording segments shown at higher temporal resolution (bottom). (I and J) (I) Sample trace of changes in O_2_ concentration associated with the switch from (a) glucose (10 mM) to (b) lactate (20 mM) (ambient atmosphere with 20% oxygen, O_2_ microsensor placed at 50 μm depth) over time (*t*), and (J) respective quantification of the O_2_ concentration (O_2_). *n/N* (slices/preparations): 14/4. ∗p < 0.05, paired t test. Note that in lactate the O_2_ concentration decreases despite the attenuation of gamma oscillations (5H and 5I). Note that the experiments were done in *ex vivo* slices (A–G) and slice cultures (H–J), with different recording conditions. Data are given as median ± interquartile range (IQR = 75% percentile - 25% percentile), error bars indicate minimal and maximal values.

The switch from sharp wave-ripples to highly synchronized gamma oscillations in *ex vivo* slices and in standard glucose (10 mM) markedly decreased the tissue O_2_ concentration (Figures 5A, 5B, and 5D), indicative of increased oxidative ATP synthesis. To obtain a more precise estimate of the cerebral metabolic rate of oxygen (CMRO_2_), we used oxygen depth profiles with high spatial resolution (Figure 5C) and mathematical modeling of convective transport, diffusion, and activity-dependent consumption of oxygen (Schneider et al., 2019). Indeed, CMRO_2_ was about 1.5-fold higher during highly synchronized gamma oscillations in the presence of glucose (Figure 5E).

Lactate (20 mM) markedly decreased the tissue O_2_ concentration and increased the CMRO_2_ by about 9% during sharp wave-ripples (Figures 5F and 5G), with no change in the level of network activity (Figure 4). Similar results were obtained with lactate (20 mM) during less synchronized gamma oscillations in slice cultures at ambient normoxia (Figure 5J). Notably, the decrease in tissue O_2_ concentration coincided with a clear attenuation of gamma oscillations (Figures 5H, 5I, and 3A–3G). This paradoxical effect of lactate hampered precise estimates on lactate-induced increases in CMRO_2_, however.

These data show that sharp wave-ripples feature lower energy expenditure than gamma oscillations and that lactate increases the oxygen consumption during unchanged or even attenuated network rhythms.

### Lactate Attenuates Excitatory and Inhibitory Synaptic Transmission

**Figure 6:**
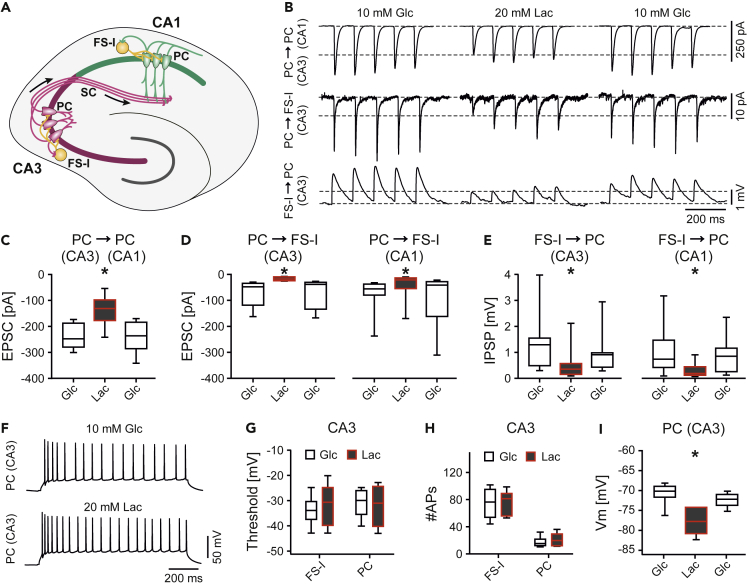
Lactate-Evoked Attenuation of Synaptic Transmission Paired patch-clamp recordings were done in CA3 and CA1 in *ex vivo* slices. (A) Scheme to illustrate the electrical stimulations (10 Hz) shown in (B) and summarized in (C)–(E). Excitatory postsynaptic currents (EPSCs) in pyramidal cells (PC, green) of CA1 were evoked by extracellular stimulation of the Schaffer Collaterals (SC) formed by axons of pyramidal cells in CA3 (PC, dark magenta). EPSCs in fast-spiking interneurons (FS-I, yellow) of CA1 and CA3 were evoked by extracellular stimulation of excitatory inputs from pyramidal cells. Inhibitory postsynaptic potentials (IPSPs) in pyramidal cells of CA1 and CA3 were evoked by triggering action potentials in synaptically connected fast-spiking interneurons. (B) Sample traces of postsynaptic responses (average from 50 to 100 sweeps of electrical stimulation) in pyramidal cells (top and bottom) or in fast-spiking interneurons (middle). Responses were evoked in glucose (Glc, 10 mM) or in lactate (Lac, 20 mM). (C) Quantification of evoked EPSCs for CA1. *n/N* (cells/animals): PC (CA3) → PC (CA1), 6/6. Each ∗p < 0.05 versus Glc (prior to and after lactate), One-way ANOVA with Holm-Šídák's multiple comparisons test. (D) Quantification of evoked EPSCs for CA3 (left) and CA1 (right). *n/N* (cells/animals): PC → FS-I (CA3), 8/7; PC → FS-I (CA1), 6/6. Each ∗p < 0.05 versus Glc (prior to and after lactate), Friedman with Tukey's pairwise comparisons test. (E) Quantification of evoked IPSPs in PCs of CA3 (left) and CA1 (right). *n/N*: FS-I → PC (CA3), 9/8; FS-I → PC (CA1), 9/9. Each ∗p < 0.05 versus Glc (prior to and after lactate), Friedman with Tukey's pairwise comparisons test. (F) Sample traces of electrical responses in PCs in CA3 evoked by depolarizing current injection (300 pA) in glucose (Glc, 10 mM) (top) or lactate (Lac, 20 mM) (bottom). (G–I) Neurons in CA3 were analyzed for different parameters. *n/N* (cells/animals): FS-I (CA3), 6/4; PC (CA3), 6/3. (G) Threshold of action potential generation (Threshold). Mann-Whitney test. (H) Number of action potentials (#APs). Mann-Whitney test. (I) Resting membrane potential (Vm) recorded from pyramidal cells (PC). Each ∗p < 0.05 versus Glc (prior to and after lactate), Friedman with Tukey's pairwise comparisons test. Note that stimulation artifacts were removed from the sample traces (B). Data are given as median ± interquartile range (IQR = 75% percentile - 25% percentile), error bars indicate minimal and maximal values. See also Figure S3.

Gamma oscillations and sharp wave-ripples are generated by precise mutual synaptic transmission between excitatory pyramidal cells and fast-spiking, GABAergic interneurons that inhibit the perisomatic region of pyramidal cells (Hájos and Paulsen, 2009; Schlingloff et al., 2014; Colgin, 2016). Notably, these interneurons show unique electrophysiological properties associated with high energy demand (Kann, 2016). To further examine the cellular mechanisms through which lactate disturbs fast network oscillations, we performed single and paired patch-clamp recordings in excitatory pyramidal cells and fast-spiking, inhibitory interneurons (Figure 6A) in two regions of the hippocampus, i.e., CA3 and CA1 (Rozov et al., 2001; Valiullina et al., 2017).

We first characterized the excitatory drive that pyramidal cells (Figures 6B, top, and 6C) and fast-spiking interneurons (Figures 6B, middle, and 6D) receive at their dendrites in standard glucose (10 mM) and in lactate (20 mM), combining whole-cell, voltage-clamp recordings with extracellular electrical stimulation. To improve the “space-clamp” condition, we used Cs-containing intracellular solution. Lactate generally attenuated evoked excitatory postsynaptic currents (EPSCs) in pyramidal cells and in fast-spiking interneurons, which was reversible (Figures 6C and 6D). Next, we determined the efficacy of synaptic transmission from fast-spiking interneurons to connected (postsynaptic) pyramidal cells using paired patch-clamp recordings (Figures 6B, bottom, and 6E). Although lactate did not affect the generation of action potentials in the axon of interneurons, it attenuated perisomatic inhibitory postsynaptic potentials (IPSPs) in pyramidal cells, which was reversible (Figure 6E). These experiments were done with 10-Hz stimulation in either type of synapse.

Despite these clear effects on synaptic transmission, lactate affected neither the threshold for the generation of action potentials (“spiking”) (Figures 6F and 6G) nor the number of action potentials (Figure 6H) as revealed by intracellular injection of depolarizing electrical currents in pyramidal cells and fast-spiking, inhibitory interneurons (see also Figure S3). The slightly hyperpolarized resting membrane potentials (Figure 6I) primarily reflect the lowered external NaCl concentration (Transparent Methods: Electrophysiology). Thus, the neurons recorded in the upper layers of the slice basically tolerated sole lactate (20 mM), which can alter the metabolic state of the cells by shifting intracellular osmotic balance, NAD^+^/NADH and lactate/pyruvate ratios.

These data show that sole lactate attenuates synaptic transmission, whereas membrane excitability and spiking properties of different types of neurons are overall regular.

### Lactate Reduces the Neurotransmitter Content at Excitatory and Inhibitory Synapses

**Figure 7:**
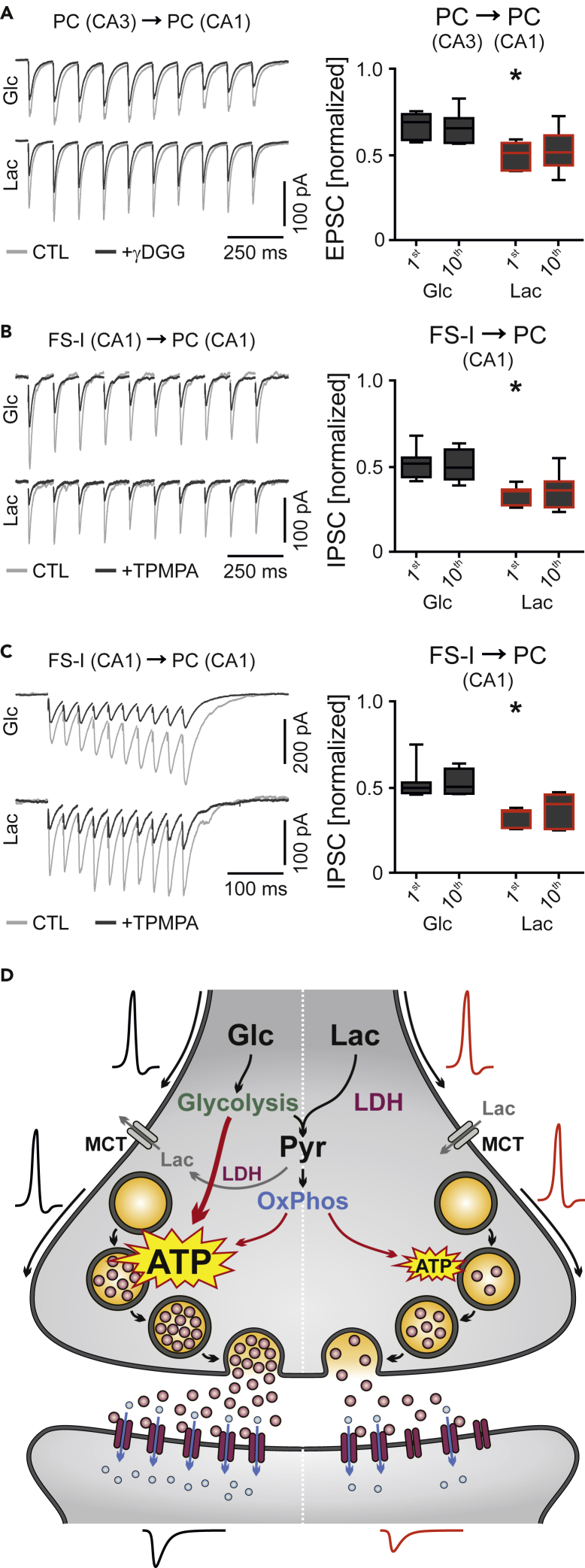
Lactate-Evoked Reduction in Transmitter Release Patch-clamp recordings were done in CA1 in *ex vivo* slices. Postsynaptic currents were isolated and partially blocked with low-affinity competitive antagonists (Transparent Methods: Electrophysiology). (A, left) Sample traces (light gray) of excitatory postsynaptic currents (EPSCs) in CA1 pyramidal cells (PC) evoked by extracellular electrical stimulation (10 Hz) of the Schaffer Collaterals in glucose (Glc, 10 mM, upper panel) or in lactate (Lac, 20 mM, lower panel) as control (CTL) conditions. Application of gamma-D-glutamylglycine (γDGG, 0.5 mM) (dark gray traces) revealed the stronger reduction of AMPA receptor-mediated excitation in Lac. (A, right) Quantification of the first and the tenth evoked EPSC in Glc or in Lac (γDGG normalized to control). *n/N* (cells/animals): Glc, 6/6; Lac, 6/6. ∗p < 0.05 versus first Glc, unpaired t test. (B and C, left) Sample traces (light gray) of inhibitory postsynaptic currents (IPSCs) in CA1 pyramidal cells (PC) evoked by extracellular electrical stimulation (10 and 40 Hz) of inputs from fast-spiking interneurons (FS-I) in glucose (Glc, 10 mM, upper panel) or in lactate (Lac, 20 mM, lower panel) as control (CTL) conditions. Application of (1,2,5,6-Tetrahydropyridin-4-yl)methylphosphinic acid (TPMPA, 200 μM) (dark gray traces) revealed the larger suppression of GABA_A_ receptor-mediated inhibition in Lac at 10 Hz (B) and at 40 Hz (C). (B and C, right) Quantification of the first and the tenth evoked IPSC in Glc or in Lac (TPMPA normalized to control). *n/N* (cells/animals): Glc, 8/8; Lac, 7/7. ∗p < 0.05 versus first Glc, unpaired t test (B) and Mann-Whitney test (C). Note that stimulation artifacts were removed from the sample traces (A–C). Data are given as median ± interquartile range (IQR = 75% percentile - 25% percentile), error bars indicate minimal and maximal values. (D) Proposed mechanism for lactate-evoked attenuation of synaptic transmission. Left, the degradation of glucose (Glc) to pyruvate (Pyr) during glycolysis results in fast ATP supply for vesicle filling with neurotransmitter, which is supported by ATP synthesis through oxidative phosphorylation (OxPhos) in mitochondria. Pyr is partially converted to lactate (Lac) through the lactate dehydrogenase (LDH), which then leaves the presynaptic terminal through monocarboxylate transporters (MCT) along the concentration gradient. Right, sole Lac results in the production of Pyr through the LDH. Pyr is further metabolized by mitochondrial OxPhos for slow ATP supply. The omission of fast glycolytic ATP supply causes reduced neurotransmitter content in presynaptic vesicles and thus smaller postsynaptic responses at excitatory and inhibitory synapses.

The filling of neurotransmitters into vesicles in presynaptic terminals is an active process that requires substantial amounts of ATP to power the vacuolar H^+^-ATPase (Ashrafi and Ryan, 2017; Dienel, 2019). To further identify the mechanism by which lactate attenuates synaptic transmission, we explored the neurotransmitter content at active synapses. For this purpose, we investigated AMPA receptor-mediated glutamatergic transmission at excitatory synapses and GABA_A_ receptor-mediated transmission at inhibitory synapses by combining extracellular stimulation, patch-clamp recordings and pharmacology to isolate and partially block the respective postsynaptic currents (Figure 7 and Transparent Methods: Electrophysiology).

We first explored the synaptic glutamate content by eliciting EPSCs in pyramidal cells with electrical synaptic stimulation (10 Hz) in the presence of the low-affinity competitive AMPA receptor antagonist γ-D-glutamylglycine (γDGG) (Liu et al., 1999; Watanabe et al., 2005). Application of γDGG reduced the amplitudes of EPSCs during electrical stimulation in the presence of either glucose (10 mM) or lactate (20 mM) (Figure 7A). However, the effect of γDGG was significantly larger in lactate.

Next we explored the synaptic GABA content by eliciting inhibitory postsynaptic currents (IPSCs) in pyramidal cells with electrical extrasynaptic stimulation (10 and 40 Hz) in the presence of the low-affinity competitive GABA_A_ receptor antagonist (1,2,5,6-Tetrahydropyridin-4-yl)methylphosphinic acid (TPMPA) (Jones et al., 2001). Application of TPMPA reduced the amplitudes of IPSCs during electrical stimulation in the presence of either glucose or lactate (Figures 7B and 7C). Similar to the above results, the effect of TPMPA was significantly larger in lactate. The stimulation frequency had no effect on the amplitudes of IPSCs in each condition.

The larger suppression of postsynaptic responses in the presence of low-affinity competitive receptor antagonists suggests that sole lactate generally reduces neurotransmitter release from presynaptic terminals, most likely by omitting fast glycolytic ATP supply (Figure 7D).

## Discussion

### Gamma Oscillations and Sharp Wave-Ripples

Gamma oscillations emerge in many cortical areas and mainly in awake mammals, including humans. They support action potential timing and synaptic plasticity and associate with higher brain functions, such as sensory perception, attentional selection, motor activity, and memory formation (Melloni et al., 2007; van Vugt et al., 2010; Colgin, 2016). Sharp wave-ripples arise in the hippocampus during waking immobility, consummatory behavior, and slow-wave sleep (Buzsáki, 2015; Ramirez-Villegas et al., 2015). They assist in transferring compressed hippocampal information to distributed neocortical circuits to support memory consolidation (Buzsáki, 2015; Colgin, 2016). In the present study, we induced these cortical rhythms in *ex vivo* slices and slice cultures and investigated the functional consequences of the energy substrate lactate. Slice preparations have provided important information about the cellular, synaptic, and neuromodulatory mechanisms underlying gamma oscillations and sharp wave-ripples (Mann and Paulsen, 2007; Buzsáki and Wang, 2012; Buzsáki, 2015; Butler et al., 2018; Müller and Remy, 2018). We note that slice preparations are complementary to other models, such as certain types of neurons in monoculture or in co-culture with astrocytes, featuring much less developed neuronal networks (Bouzier-Sore et al., 2003; Kann and Kovács, 2007; Baeza-Lehnert et al., 2019; Ashrafi et al., 2020). The latter models permit tight experimental control over delivery of oxygen and energy substrates (short diffusion distances, large extracellular space etc.), whereas slice preparations permit investigations of the bioenergetics underlying cortical network rhythms.

Gamma oscillations and sharp wave-ripples rely on precise mutual synaptic transmission between excitatory pyramidal cells and GABAergic interneurons. In general, pyramidal cells provide excitatory drive to interneurons that, in turn, transiently inhibit the perisomatic region of pyramidal cells through rhythmic GABA release. The inhibitory GABAergic currents largely contribute to the local field potential during gamma oscillations (Mann et al., 2005; Gulyás et al., 2010; Oren et al., 2010) and sharp wave-ripples (Schlingloff et al., 2014; Schönberger et al., 2014; Bazelot et al., 2016) in stratum pyramidale that consists of densely packed somata of pyramidal cells. Therefore, local field potential recordings primarily reflect the strongly dominating inhibitory input to the perisomatic region of pyramidal cells by GABAergic interneurons (Freund and Buzsáki, 1996; Megías et al., 2001; Wittner et al., 2007; Schneider et al., 2015). The synaptic mechanisms underlying the generation of gamma oscillations and sharp wave-ripples likely differ, however (Schlingloff et al., 2014; Donoso et al., 2018). Nevertheless, both oscillation types strongly depend on the appropriate release of neurotransmitters that requires efficient presynaptic ATP synthesis (Kann, 2016; Dienel, 2019).

Fast-spiking interneurons — prototype is the parvalbumin-positive, GABAergic basket cell — have a prominent role in rhythmic perisomatic inhibition (Hájos and Paulsen, 2009; Kann, 2016). These interneurons feature unique action potential kinetics and membrane ion currents as well as presynaptic GABA release at high rates up to >100 Hz (Gulyás et al., 2010; Hu et al., 2018). Accordingly, the neural ultrastructure is enriched with mitochondria and cytochrome *c* oxidase, indicating the importance of oxidative ATP supply (Gulyás et al., 2006; Kann, 2016). Therefore, it is likely that the disturbances of fast network rhythms, especially of persistent gamma oscillations, are mainly caused by fast-spiking interneurons rather than pyramidal cells.

Using reliable estimations of CMRO_2_, we show that gamma oscillations associate with significantly higher energy expenditure than sharp wave-ripples; similar data were recently reported for the mouse (Schneider et al., 2019). Notably, gamma and theta-gamma oscillations require a hemodynamic response to match oxygen and energy substrate demands (Niessing et al., 2005; Schneider et al., 2019).

### Lactate and Brain Energy Metabolism

Lactate is an alternative energy substrate in the brain (Hyder et al., 2006; Magistretti and Allaman, 2015; Yellen, 2018; Dienel, 2019). It can be shuttled across the membranes of brain cells through different MCTs and along the local concentration gradient (Magistretti and Allaman, 2015; Saab and Nave, 2017; Dienel, 2019).

Lactate can be generated and released from astrocytes and oligodendrocytes that primarily enwrap synapses and axons, respectively (Pellerin and Magistretti, 1994; Caesar et al., 2008; Gandhi et al., 2009; Saab et al., 2016). However, neurons can also release some lactate depending on the experimental condition (Waagepetersen et al., 2000; Dienel, 2019). Lactate uptake in neurons requires MCT-2 that has a lower Km value (∼0.7 mM) compared with the astrocytic transporters MCT-1 and MCT-4 (∼3.5 and ∼30 mM, respectively) (Hertz and Dienel, 2005). Lactate is also taken up from the blood during physical activity; exhaustive exercise, for example, can increase the arterial plasma lactate levels from about 1 mM to as high as 20 mM (Boumezbeur et al., 2010; Rasmussen et al., 2010; Dienel, 2012). High parenchymal lactate can also result from ischemia, epileptic seizures and traumatic brain injury, various systemic disorders featuring hypoglycemia and lactic acidosis, as well as therapeutic supplementation (Kraut and Madias, 2014; Magistretti and Allaman, 2015; Brooks, 2018; Dienel, 2019).

We note that the neuronal MCT-2 is proton-coupled (Halestrap, 2013). Thus, increased utilization of lactate may result in intracellular acidification (Dienel, 2017). Indeed, previous studies in neurons showed decreases in the intracellular pH by about 0.02 and 0.1 units at 5 and 20 mM lactate, respectively (Munsch and Pape, 1999; Ruusuvuori et al., 2010). Our data argue against a significant and functionally relevant intracellular acidification because of the presence of sharp wave-ripples and regular membrane excitability and spiking properties of different types of neurons in sole lactate (20 mM). However, quantification of local acidification in distinct subcellular compartments, such as presynaptic endings, in active excitatory and inhibitory neurons is technically challenging (Willoughby and Schwiening, 2002; Holmgren et al., 2010; Ruusuvuori et al., 2010).

During gamma oscillations, which feature particularly high energy expenditure, the consumption of extracellular lactate in neurons might be limited at the level of LDH-1 that converts lactate into pyruvate (Magistretti and Allaman, 2015; Dienel, 2019). Although the conversion from lactate to pyruvate is an equilibrative reaction (lactate + NAD^+^ ↔ pyruvate + NADH + H^+^), it also depends on the regeneration of cytosolic NAD^+^. This putative rate-limiting step is governed by the malate-aspartate shuttle (Brand and Chappell, 1974; Mintun et al., 2004).

### Effects on Fast Network Rhythms, Synaptic Transmission, and Oxygen Metabolism

Lactate disturbed gamma and theta-gamma oscillations in *ex vivo* slices and slice cultures under well-defined experimental conditions (Transparent Methods), similar to previous studies on metabolism and evoked synaptic activity (Cox and Bachelard, 1988; Kanatani et al., 1995). These findings do not support other reports suggesting that lactate is a full — and even preferred — energy substrate of active neurons *in vitro* and *in vivo* (Schurr et al., 1988; Izumi et al., 1997; Bouzier-Sore et al., 2003; Suzuki et al., 2011; Wyss et al., 2011). The main reasons for these diverging findings might be the use of immature neuronal cultures, the type of artificial electrical stimulation and/or anesthesia in the other reports, as well as the high energy expenditure of gamma rhythms (Niessing et al., 2005; Kann et al., 2011; Schneider et al., 2019). Remarkably, sole lactate induced recurrent bursts in *ex vivo* slices and attenuation of the oscillations in slice cultures, which might reflect general differences in the excitation-inhibition balance in both preparations. The neuronal disturbances might contribute to the (patho)physiological mechanisms underlying several (clinical) symptoms associated with high lactate levels, ranging from central fatigue during exhaustive exercise to epileptic seizures (Rasmussen et al., 2010; Kraut and Madias, 2014).

By contrast, lactate did not affect sharp wave-ripples. This finding is in line with previous reports suggesting that lactate can serve as an alternative energy substrate to support neuronal survival and basic forms of activity (Schurr et al., 1988; Izumi et al., 1997; Brown et al., 2001; Bouzier-Sore et al., 2003). The persistence of sharp wave-ripples might primarily reflect the lower energy expenditure and the intermittent nature of the events, both of which facilitate local diffusion of energy substrates and ATP and, therefore, metabolic recovery (Harris et al., 2012; Schlingloff et al., 2014; Dienel, 2019; Schneider et al., 2019). The persistence of sharp wave-ripples as well as the similar effects of low glucose and lactate on gamma oscillations argue against a significant action of the G_i_ protein-coupled receptor for lactate (HCAR1) that decreases neuronal activity *in vitro* (Bozzo et al., 2013; Dienel, 2019).

Lactate evoked recurrent neural bursts during highly synchronized gamma oscillations in *ex vivo* slices, suggesting an excitation-inhibition imbalance. Indeed, lactate generally attenuated synaptic transmission at glutamatergic (pyramidal cell → pyramidal cell, pyramidal cell → fast-spiking interneuron; dendritic excitation) and inhibitory (fast-spiking interneuron → pyramidal cell; perisomatic inhibition) synapses because of reduced neurotransmitter release. During gamma oscillations pyramidal cells sparsely generate action potentials at 1–3 Hz, whereas fast-spiking, GABAergic interneurons fire much higher at >20 Hz (Gulyás et al., 2010; Kann, 2016). Therefore, the synaptic effects of lactate that we observed during experimental electrical stimulation might differ more strongly at glutamatergic and GABAergic synapses during gamma oscillations. Gamma and theta-gamma oscillations in slice cultures maintained the excitation-inhibition balance, likely because of less synchronization and/or partial adaptation to uptake and utilization of lactate at this stage of tissue development (Limitations of the Study) (Schousboe et al., 1993; Wada et al., 1997; Dienel, 2019). We note that our slice cultures maturated in culture medium containing about 4 mM glucose (Transparent Methods: Preparation of slice cultures), which might have reduced functional alterations discussed for dissociated neuronal cultures grown in up to 30 mM glucose (Dienel, 2019). We limited the use of higher glucose and lactate concentrations to the short period of experimental recordings. However, lactate clearly attenuated gamma and theta-gamma oscillations, likely reflecting the attenuated synaptic transmission at excitatory and inhibitory synapses.

By contrast, sole lactate did not affect the intrinsic electrophysiological properties of neurons, including action potential generation. Similar findings were reported for stimulus-evoked axonal population responses (Walz and Harold, 1990; Yamane et al., 2000). These observations argue against the presence of general metabolic stress and/or acidification (see above), at least, in the soma, the proximal dendrites, and the axon. In fact, they support the concept that axons can be fueled with lactate from myelinating oligodendrocytes (Saab and Nave, 2017; Stedehouder et al., 2017).

The lactate-evoked disturbances of gamma-band rhythms are most likely caused by transient ATP shortages in presynaptic structures, particularly in fast-spiking inhibitory interneurons (Kann, 2016; Dienel, 2019). Presynaptic terminals of central neurons increase glucose uptake via GLUT-4 and upregulate glycolysis during sustained neuronal activity (stimulation at 10–20 Hz) (Ashrafi et al., 2017). This rapid way of glycolytic ATP supply has been proposed to significantly contribute to maintenance of the vesicle cycle, which is a major consumer of presynaptic ATP, especially when mitochondria are sparse or absent (Shepherd and Harris, 1998; Ikemoto et al., 2003; Ashrafi and Ryan, 2017). This notion is supported by our data demonstrating that adding low fractions of glucose (2 or 5 mM) to lactate supply (16 or 10 mM) progressively suppressed neural bursting. This is also in line with estimates that lactate can contribute up to ∼60% to oxidative brain metabolism, with glucose providing the rest (Boumezbeur et al., 2010; Dienel, 2019). By using pharmacological isolation and partial blockade of postsynaptic currents, we further identify the reduced neurotransmitter release from presynaptic endings of excitatory and inhibitory neurons in the presence of lactate (i.e., the lack of fast glycolytic ATP supply).

Lactate-evoked disturbances of gamma-band rhythms might also reflect limited neuronal uptake (MCT-2) and/or conversion (LDH-1) of lactate (Bak et al., 2006; Dienel, 2012), acidification of subcellular compartments (Walz and Harold, 1990; Dienel, 2019), and/or alterations of enzymes and ion channels caused by shutdown of the pentose phosphate pathway and concomitant changes in the redox state (Bolaños, 2016). However, detailed knowledge about the bioenergetic properties of dendrites, axons, and presynaptic terminals in the different types of excitatory and inhibitory neurons is lacking (Buzsáki et al., 2007; Kann, 2016; Dienel, 2019).

Lactate increased the oxygen consumption during sharp wave-ripples and gamma oscillations. For sharp wave-ripples, we calculated an increase in CMRO_2_ of about 9% under aerobic conditions; this range likely reflects enhanced oxidative metabolism in mitochondria to compensate for the shutdown of glycolytic ATP synthesis (Harris et al., 2012; Dienel, 2019). These data show that during network rhythms with lower energy expenditure, such as sharp wave-ripples, the cellular ATP synthesis can be reliably adapted, even when lactate replaces glucose.

Our finding that the lactate-induced increase in oxygen consumption can coincide with strikingly divergent effects on neuronal activity (no effects during sharp wave-ripples versus bursting or attenuation during gamma oscillations) is relevant to the interpretation of brain imaging data, such as fMRI (Magistretti and Allaman, 2015; Scheeringa and Fries, 2019).

The lactate-induced disturbances of gamma-band rhythms that we report here argue for careful therapeutic supplementation of exogenous lactate in neurologic (brain ischemia or traumatic injury) and other intensive care patients (Glenn et al., 2015; Magistretti and Allaman, 2015; Brooks, 2018).

### Conclusions

Our data establish key principles regarding lactate and its effectiveness in fueling fast brain rhythms: (1) Lactate disturbs cortical gamma rhythms by neural bursting or attenuation, whereas sharp wave-ripples featuring lower energy expenditure resist. (2) Lactate increases the oxygen consumption, whereas neuronal network activity can even decrease. (3) Lactate attenuates synaptic transmission by reduced neurotransmitter release rather than altered intrinsic neuronal membrane properties, including action potential generation, in excitatory and inhibitory neurons. These principles are relevant to the general understanding of electrical and metabolic consequences of lactate fuel in neurons in health and disease, the interpretation of functional brain imaging, and the clinical application of lactate supplementation.

### Limitations of the Study

We used *ex vivo* slices and slice cultures of the hippocampus that generally lack functional vasculature and blood supply. Therefore, slices are provided with external recording solution (artificial cerebrospinal fluid, aCSF). Slice cultures can tolerate relatively low fractions of oxygen (20%) and energy substrates (e.g., 5 mM glucose), whereas *ex vivo* slices require high oxygen (95%) and glucose (10 mM) fractions in the external recording solution to fuel gamma oscillations. However, the final concentrations of energy substrates depend on molecular properties, including weight, size, and charge, as well as diffusion, transport, and consumption within the tissue. In addition, the types of preparation (*ex vivo* slice with 400 μm thickness or slice culture with about 250 μm thickness) and the recording conditions (slice submerged in recording solution or slice kept at the interface of recording solution and ambient gas atmosphere) have a role. Because of technical limitations, we are currently unable to provide the exact concentrations of glucose and lactate for each depth of a slice. However, the large fall in the glucose concentration from the slice surface to the slice core to about one-third has been described for unstimulated hippocampal *ex vivo* slices, similar to the large falls in the O_2_ concentration.

The concentrations of glucose (10 mM) and lactate (20 mM) used in our experiments are not isocaloric. This is because oxidation of one glucose molecule yields two more ATP than oxidation of two lactate molecules due to glycolytic metabolism. In addition, the rates of diffusion, membrane transport (MCTs), and conversion to pyruvate (LDH-1) might contribute to substantial differences between lactate and glucose metabolism.

We used *ex vivo* hippocampal slices from the young adult rat as well as organotypic hippocampal slice cultures from the rat pup after 10–15 days *in vitro*, which roughly corresponds to the third and fourth postnatal week *in vivo*. Whether our findings also apply to other species, cortical regions, and developmental stages needs to be addressed in further studies.

Our study on the effects of sole lactate fuel on cortical network rhythms and electrophysiological properties of central excitatory and inhibitory neurons is a complementary experimental approach to provide further insights into brain energy metabolism, without favoring either side of the controversy about the general role of lactate. Here, we demonstrate that sole lactate can fuel sharp wave-ripples. However, lactate is supplemental to some obligatory glucose in fueling gamma oscillations that feature high energy expenditure.

### Resource Availability

#### Lead Contact

Further information and requests for resources and reagents should be directed to and will be fulfilled by the Lead Contact, Oliver Kann (oliver.kann@physiologie.uni-heidelberg.de).

#### Materials Availability

This study did not generate new unique reagents.

#### Data and Code Availability

The datasets have not been deposited in a public repository because of the large size of electrophysiological time-series data. They are available from the Lead Contact on request. This study did not generate codes.

## Methods

All methods can be found in the accompanying Transparent Methods supplemental file.

## Supplemental Information

Document S1. Transparent Methods, Figures S1–S3, and Table S1
