# Supplementary material for: Lactate Attenuates Synaptic Transmission and Affects Brain Rhythms Featuring High Energy Expenditure
Source: iScience. 2020 Jun 27;23(7):101316. doi: 10.1016/j.isci.2020.101316 (PMC7350153; doi:10.1016/j.isci.2020.101316)
Supplement: Document S1. Transparent Methods, Figures S1–S3, and Table S1 [file mmc1.pdf]

iScience, Volume *23*

Supplemental Information

Lactate Attenuates Synaptic

Transmission and Affects Brain Rhythms

Featuring High Energy Expenditure

Jan-Oliver Hollnagel, Tiziana Cesetti, Justus Schneider, Alina Vazetdinova, Fliza Valiullina-Rakhmatullina, Andrea Lewen, Andrei Rozov, and Oliver Kann

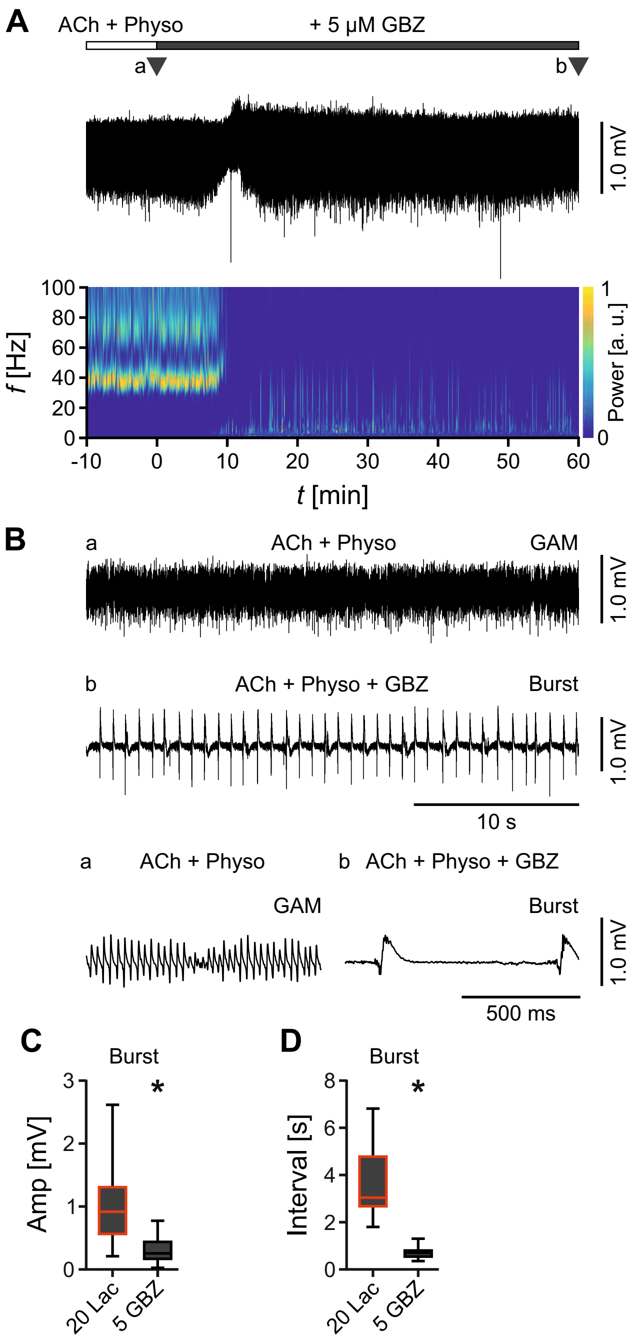

Figure S1. Block of inhibition replaces gamma oscillations with neural bursts, Related to Figure 2.
Local field potentials were recorded in stratum pyramidale of CA3 in *ex vivo* slices; gamma oscillations (GAM) were induced by bath application of acetylcholine (ACh, 10 µM) and physostigmine (Physo, 2 µM).
(A) Sample trace for gamma oscillations in glucose (10 mM) followed by additional application of gabazine (GBZ, 5 µM) and corresponding wavelet transformation showing power of frequency domains (f) over time (t). Heat-scale colors encode for power (Power) in arbitrary units (a.u.). Triangles (a, b) indicate recording segments shown at higher temporal resolution in (B).
(B) Sample traces of (a) gamma oscillations and (b) neural bursting activity in presence of GBZ.
(C-D) Neural bursts were analyzed for amplitude (Amp) and interval (Interval). *n/N* (slices/animals): 20 Lac, 17/9; 5 GBZ, 20/4. The onset of bursting activity was around 37.4 ± 4 min (20 Lac, mean ± SEM) and significantly faster when GBZ was applied 10.5 ± 1 min (5 GBZ, mean ± SEM). $^*p < 0.05$, Mann-Whitney test. (C) Amplitude of bursts (Amp) in CA3. $^*p < 0.05$, Mann-Whitney test. (D) Interval of bursts (Interval) in CA3. $^*p < 0.05$, Mann-Whitney test.
Note the absence of gamma oscillations between neural bursts. Data are given as median ± interquartile range (IQR = 75% percentile - 25% percentile), error bars indicate minimal and maximal values.

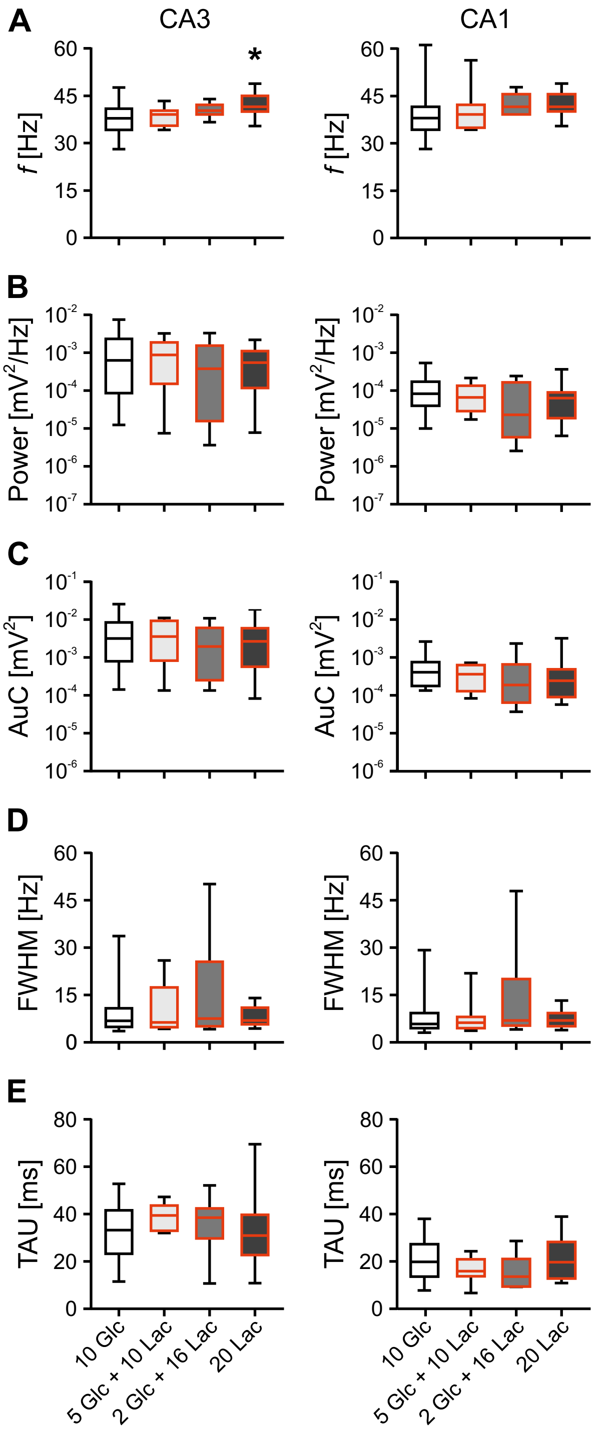

Figure S2. Gamma oscillations fueled by metabolites in different combinations are similar, Related to Figure 2.
Local field potentials were recorded in stratum pyramidale of CA3 (left) and CA1 (right) in *ex vivo* slices; different combinations of glucose (Glc) and lactate (Lac) were applied following successful induction of gamma oscillations (GAM) by bath application of acetylcholine (10 µM) and physostigmine (2 µM).
(A-E) Gamma oscillations were analyzed for different parameters. *n/N* (slices/animals): CA3, 10 Glc, 48/16; CA1, 10 Glc, 31/11; 5 Glc + 10 Lac, 8/4; 2 Glc + 16 Lac, 9/5; 20 Lac, 14/8. (A) Peak frequency (f). CA3, *$p < 0.05$ vs 10 Glc, one-way ANOVA with Holm-Šídák's multiple comparisons test. CA1, Kruskall-Wallis with Dunn's multiple comparisons test. (B) Peak of power spectral density (Power). CA3 & CA1, Kruskall-Wallis with Dunn's multiple comparisons test. (C) Area under the curve (AuC). CA3 & CA1, Kruskall-Wallis with Dunn's multiple comparisons test. (D) Full width at half maximum (FWHM) derived from power spectra. CA3 & CA1, Kruskall-Wallis with Dunn's multiple comparisons test. (E) Time constant (TAU) of the decaying exponential fit to the peaks of the autocorrelation. CA3 & CA1, Kruskall-Wallis with Dunn's multiple comparisons test.
Data are given as median ± interquartile range (IQR = 75% percentile - 25% percentile), error bars indicate minimal and maximal values.

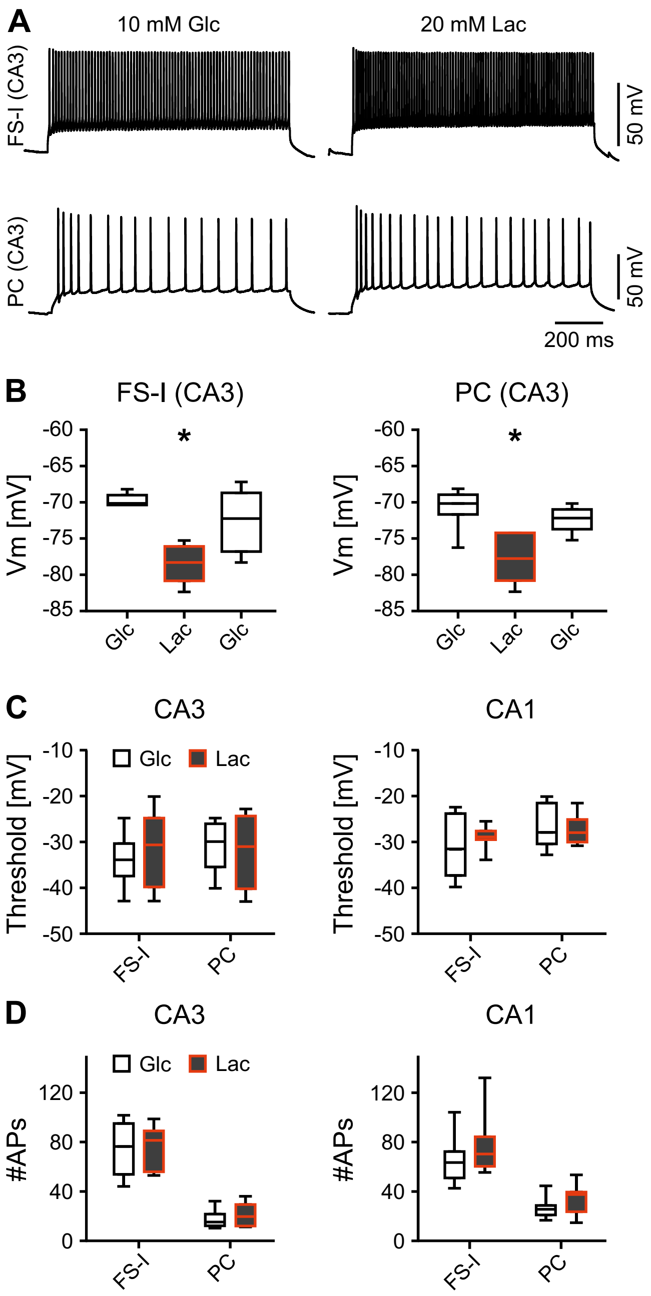

Figure S3. Intrinsic membrane properties of inhibitory and excitatory neurons in *ex vivo* slices, Related to Figure 6.
Patch-clamp recordings were done in CA3 and CA1 in *ex vivo* slices.
(A) Sample traces of electrical responses in fast-spiking interneurons (FS-I) (top) and pyramidal cells (PC) (bottom) in CA3 evoked by depolarizing current injection (300 pA) in glucose (Glc, 10 mM) or lactate (Lac, 20 mM).
(B-D) Neurons were analyzed for different parameters. *n/N* (cells/animals): FS-I (CA3), 6/4; FS-I (CA1), 8/4; PC (CA3), 6/3; PC (CA1), 7/4. (B) Resting membrane potential (Vm) recorded from fast-spiking interneurons (FS-I) (left) and pyramidal cells (PC) (right). Each *$p < 0.05$ vs Glc (prior to and after lactate), Friedman with Tukey's pairwise comparisons test. (C) Threshold of action potential generation (Threshold). Mann-Whitney test. (D) Number of action potentials (#AP). Mann-Whitney test. Note the much lower spiking rates of excitatory pyramidal cells. Data are given as median ± interquartile range (IQR = 75% percentile - 25% percentile), error bars indicate minimal and maximal values.

Transparent Methods

Table S1: Key Resources

REAGENT or RESOURCE	SOURCE	IDENTIFIER
Bacterial and Virus Strains		
AAV-CaMKIIα-hChR2(H134R)-mCherry	UNC Gene Therapy Center Vector Core	N/A
Chemicals, Peptides, and Recombinant Proteins		
Acetylcholine	Sigma-Aldrich	CAT#A6625; CAS: 60-31-1
Physostigmine	Tocris	CAT#0622; CAS: 64-47-1
Na-L-Lactate	Alfa-Aesar	CAT#L14500; CAS: 867-56-1
D-APV	Tocris	CAT#0106; CAS: 79055-68-8
SR-95531 (Gabazine)	Sigma-Aldrich	CAT#S106; CAS: 104104-50-9
γDGG	Tocris	CAT#0112; CAS: 6729-55-1
Cyclothiazide	Tocris	CAT#0713; CAS: 2259-96-3
CNQX	Tocris	CAT#0190; CAS: 115066-14-3
CGP 54626	Tocris	CAT#1088; CAS: 149184-21-4
CP 55,940	Sigma-Aldrich	CAT#C1112; CAS: 8302-04-4
TPMPA	Tocris	CAT#1040; CAS: 182485-36-5
Experimental Models: Organisms/Strains		
Rat	Charles-River Laboratories	Wistar
Rat	Breeding facilities of the Kazan Federal University	Wistar
Software and Algorithms		
MATLAB	Mathworks	https://www.mathworks.com/products/matlab.html
Prism	GraphPad	https://www.graphpad.com/scientific-software/prism/
Spike2	Cambridge Electronic Design	http://ced.co.uk/products/spkovin
Patchmaster	HEKA Elektronik	https://www.heka.com/downloads/downloads_main.html
IGOR PRO	WaveMetrics	https://www.wavemetrics.com/downloads/current
SigmaPlot	Systat Software GmbH	http://www.systat.de/downloads.html
SensorTrace	Unisense A/S	https://www.unisense.com/Software_download/

Experimental model and subject details

Experiments were performed in organotypic hippocampal slice cultures (Kann et al., 2011) and in *ex vivo* (acute) hippocampal slice preparations from male Wistar rats (source: Charles-River Laboratories and in-house breeding facilities of the Kazan Federal University). All animal procedures were performed in accordance with the guidelines of the European Commission and were approved by the regional authorities of Baden-Württemberg (T46/14, T96/15, and T45/18) and the Kazan Federal University regulations on the use of laboratory animals (ethical approval by the Institutional Animal Care and Use Committee of Kazan State Medical University N9–2013).

Preparation of *ex vivo* slices

Adult Wistar rats (aged 6 - 8 weeks, ~200 g) were decapitated during isoflurane anesthesia (1.5 vol% of isoflurane in a gas mixture of 70% N_2O and 30% O_2). Brains were rapidly removed and immediately transferred to aCSF (see below) at ~4°C, saturated with 95% O_2 and 5% CO_2. Horizontal hippocampal slices with 400 µm thickness were prepared at an angle of about 12.5° in the fronto-occipital direction (with the frontal portion up) using a Leica VT1000S Vibratome (Wetzlar, Germany) (Behrens et al., 2005). This orientation preserves the connectivity within hippocampal regions as well as to the entorhinal cortex. After cutting, slices were immediately transferred to a Haas-type interface recording chamber, perfused with aCSF at a flow rate of 1.8 ml/min and maintained at 34 ± 1°C. Recordings were started after 2 h of recovery. For patch-clamp recordings, horizontal hippocampal slices with 300 µm thickness were prepared from 3 - 4 week-old Wistar rats and stored until experiments at room temperature (22 - 24°C).

Preparation of slice cultures

Organotypic slice cultures were prepared as follows (Kann et al., 2011; Huchzermeyer et al., 2013): hippocampal slices (400 µm) were cut with a McIlwain tissue chopper (Mickle Laboratory Engineering Company Ltd., Guildford, UK) from 9 - 10 days-old Wistar rats (Charles-River, Sulzfeld, Germany) under sterile conditions. Slices were maintained on Biopore™ membranes (Millicell standing inserts, Merck Millipore, Darmstadt, Germany) between culture medium, consisting of 50% minimal essential medium, 25% Hank's balanced salt solution (Sigma-Aldrich, Taufkirchen, Germany), 25% horse serum (Life Technologies, Darmstadt, Germany), and 2 mM L-glutamine (Life Technologies), kept at pH 7.3, and humidified normal atmosphere (5% CO_2, 36.5°C) in an incubator (Heracell, Thermoscientific, Dreieich, Germany). The calculated glucose concentration in the culture medium was about 4 mM. Using this glucose concentration in slice cultures, we aimed to reduce long-term adaptations in the expression of metabolic enzymes that have been discussed for cultures of primary neurons and astrocytes maintained in the presence of high glucose (Kann and Kovács, 2007; Dienel, 2017; Dienel, 2019).The culture medium (1 ml) was replaced three times a week. Slice cultures were used after 10 - 15 days *in vitro* (DIV) (residual thickness of about 250 µm), when the tissue had recovered from the slice preparation and damaged cut surfaces were reorganized (Kann and Kovács, 2007). For recordings, the intact Biopore™ membrane carrying slice cultures was inserted into the interface type recording chamber (Huchzermeyer et al., 2013). Slice cultures were maintained at the interface between artificial cerebrospinal fluid (aCSF, flow rate 1.8 ml/min) and ambient gas mixture (75% N_2, 20% O_2 and 5% CO_2, flow rate 1.5 l/min). Intact Biopore™ membrane inserts ensure constant supply of oxygen and energy substrates from the recording solution that flows underneath the Biopore™ membrane; the interface condition permits constant oxygen supply from the ambient gas mixture.

Notably, hippocampal slices mature during the culture period (Bahr et al., 1995; De Simoni et al., 2003). With respect to the animal's age at preparation, slice cultures at 10 - 15 DIV feature complex networks of interconnected pyramidal cells and interneurons in the presence of glial cells (Schneider et al., 2015). The absence of hyperexcitable network states such as neural bursts indicates a well-balanced interplay between neuronal excitation and inhibition. This is crucial for reliable induction of network activities like gamma oscillations that are highly dependent on precise timing of action potentials.

Recording solution and drugs

Slice cultures as well as *ex vivo* slices were constantly supplied with warmed (34 ± 1°C) aCSF that contained (in mM): 129 NaCl, 21 $NaHCO_3$, 1.25 NaH_2PO_4, 1.8 $MgSO_4$, 1.6 $CaCl_2$, 3 KCl, 10 glucose (Sigma-Aldrich). The osmolarity was 300 ± 5 mOsmol/l and pH was 7.4 when saturated with 5% CO_2.

To facilitate the induction of sharp wave-ripples in *ex vivo* slices the concentration of $MgSO_4$ was lowered to 1.2 mM 1 h before starting the experiments (Behrens et al., 2005; Hollnagel et al., 2014). Induction of gamma oscillations was achieved by bath application of the cholinergic receptor agonist acetylcholine (*ex vivo* slices: 10 µM, and slice cultures: 2 µM; Sigma-Aldrich) and the acetylcholine-esterase inhibitor physostigmine (*ex vivo* slices: 2 µM, and slice cultures: 0.4 µM; Tocris, Bio-Techne GmbH, Wiesbaden-Nordenstadt, Germany). When substituting glucose with Na-L-lactate (Alfa-Aesar, Karlsruhe, Germany), we lowered the concentration of NaCl to 114 mM, thereby maintaining osmolarity. For pharmacological isolation of EPSCs, the following drugs were applied: D-AP5 (100 µM; NMDA receptor antagonist, Tocris), gabazine (30 µM; $GABA_A$ receptor antagonist, Sigma-Aldrich), γDGG (0.5 mM; low-affinity competitive AMPA receptor antagonist, Tocris), and cyclothiazide (100 µM; allosteric modulator reducing AMPA receptor desensitization, Sigma-Aldrich). For pharmacological isolation of IPSCs, the following drugs were applied: CNQX (10 µM; AMPA receptor antagonist, Tocris), D-AP5 (100 µM; NMDA receptor antagonist, Tocris), CGP 54626 (1 µM; $GABA_B$ receptor antagonist, Tocris), CP 55,940 (2 µM, CB receptor agonist, Sigma-Aldrich), and TPMPA (200 µM; low-affinity competitive $GABA_A$ receptor antagonist, Tocris).

Most recordings were made in a custom-built 'interface' chamber, in which hippocampal slices are maintained at the interface between the recording solution and the gas atmosphere (Haas et al., 1979; Schmitz et al., 1995; Fleidervish et al., 1996; Kann, 2012). This chamber permits excellent recovery and preservation of the cytoarchitecture for hours after the preparation. This is reflected by the presence of neuronal network rhythms, such as gamma oscillations and sharp wave-ripples, which occur in the hippocampus *in vivo* (Behrens et al., 2005; Kann et al., 2011; Schneider et al., 2019). Notably, energy substrates reach the core of *ex vivo* slices mainly through diffusion from the recording solution flowing at the slice edges and creating a thin (about 50 µm) fluid layer above the slice; this differs from 'submerged' recording conditions (Kann and Kovács, 2007; Hájos and Mody, 2009). In slice cultures, energy substrates reach the core also from flow underneath because of maintenance on Biopore membranes (Huchzermeyer et al., 2013) and thus permit experimental conditions closer to the *in vivo* situation.

Hippocampal slices have been empirically kept at elevated glucose concentrations of up to 25 mM to ensure recovery and viability during experiments in both interface and submerged recording chambers (McIlwain, 1951; Schmitz et al., 1995; Bischofberger et al., 2006; Kann et al., 2011; Kann, 2012; Hu and Jonas, 2014). The elevated glucose concentration is necessary to provide sufficient substrate supply to the slice core because slice preparations inherently lack blood flow and feature longer diffusion distances (Kann and Kovács, 2007; Schneider et al., 2019).

The elevated concentration of standard glucose (10 mM) or lactate (20 mM) is required, in particular when studying physiological neuronal network rhythms. This is because gamma oscillations, for example, feature much higher energy expenditure than the widely undefined network activities evoked by artificial electrical stimulation in slice preparations (Schurr et al., 1988; Ivanov and Zilberter, 2011; Kann et al., 2011; Hall et al., 2012; Ivanov et al., 2014; Engl et al., 2017; Schneider et al., 2019). We note that the glucose and lactate concentrations in the slice core are likely significantly lower compared with the external recording solution because of diffusion and activity-dependent consumption (Kann and Kovács, 2007). Indeed, it was recently shown that even under 'submerged' conditions, which offer better supply with energy substrates, the concentration of 10 mM glucose provided with the external aCSF resulted in a glucose concentration of about 3.1 ± 0.4 mM in the slice core (Lourenço et al., 2019). Data for lactate in similar experimental conditions is currently lacking (see also: Limitations of the Study). In addition, the usage of 95% oxygen fraction is required to provide sufficient oxygen in the core of *ex vivo* slices that show steep gradients in tissue oxygen concentration (Schmitz et al., 1995; Bischofberger et al., 2006; Kann and Kovács, 2007; Schneider et al., 2019).

Electrophysiology

Extracellular local field potentials (LFP) were recorded in alternating current (AC) mode under interface conditions with carbon fiber electrodes (Kation Scientific, Minneapolis, MN, USA) or glass electrodes (filled with aCSF) pulled from GB150F-8P borosilicate capillaries (Science Products GmbH, Hofheim, Germany) with a horizontal micropipette puller (DMZ Zeitz-Puller, Zeitz-Instruments Vertriebs GmbH, Martinsried, Germany). LFPs were amplified using an EXT 10-2F amplifier in an EPMS-07 housing (npi Electronic GmbH, Tamm, Germany), filtered at 3 kHz, digitized online at 10 kHz (CED-1401, Cambridge Electronic Design, Cambridge, UK) and stored on a computer disk with Spike2 (Cambridge Electronic Design) for offline analysis.

Induction of SPW-R complexes was achieved by a high frequency stimulation protocol, in which three tetani (100 Hz, 0.4 s) with an interval of 40 s were applied and repeated up to 6 times every 5 min (Behrens et al., 2005). The stimulus intensity was adjusted to a submaximal level of about 60 - 70% of the maximal response.

Whole-cell, patch-clamp experiments were performed under submerged conditions at 34 ± 1°C. In brief, cells were identified visually using infrared differential contrast video microscopy and by their spiking pattern in the presence of depolarizing current injections. Whole-cell recordings were performed simultaneously from two synaptically connected neurons using pipettes with resistances of 3 - 5 MΩ. Presynaptic CA3 or CA1 fast-spiking interneurons (FS-I) were stimulated with 10 Hz trains of five suprathreshold current pulses. Inhibitory postsynaptic potentials (IPSPs) were recorded from pyramidal cells (PC) in the same hippocampal region. Trains were delivered with an interval of 10 s. For recordings of excitatory postsynaptic currents (EPSCs), interneurons were held at -70 mV. To evoke synaptic currents extracellularly, theta glass electrodes filled with aCSF (Valiullina et al., 2017) were placed in stratum radiatum within ~50 - 100 µm from the soma of the recorded neuron. Inhibitory synaptic transmission during recordings was blocked by gabazine (10 µM) co-applied with the aCSF.

AMPA-mediated EPSCs were triggered in CA1 pyramidal cells by electrical stimulation of the Schaffer Collaterals. NMDA and $GABA_A$ receptor channels were blocked by bath application of D-AP5 (100 µM) and gabazine (30 µM), respectively. To test whether lactate reduces the neurotransmitter content at glutamatergic synapses, we used the low-affinity competitive AMPA receptor antagonist γDGG (Liu et al., 1999; Watanabe et al., 2005). Because of its low affinity, γDGG rapidly unbinds from AMPA receptors and permits ambient glutamate to rebind. Hence, AMPA receptor-mediated EPSCs arising from a lower relative synaptic glutamate content are inhibited more effectively by γDGG. To exclude the possible contribution of channel desensitization to EPSC amplitudes, we performed these experiments in the presence of cyclothiazide (100 µM), an allosteric modulator that reduces AMPA receptor desensitization (Partin et al., 1994).

To test whether lactate reduces the neurotransmitter content at GABAergic synapses formed by fast-spiking interneurons in the perisomatic region of pyramidal cells, we had to exclude the influence of presynaptic whole-cell dialysis on the presynaptic vesicle cycle. Consequently, we applied extracellular stimulation using a double barrel glass stimulation pipette, which was placed in stratum pyramidale near the postsynaptic pyramidal cell. Glutamatergic synaptic transmission and possible activation of $GABA_B$ receptors were blocked by application of CNQX (10 µM), D-AP5 (100 µM) and CGP 54626 (1 µM). Release from CB1-positive perisomatic terminals was abolished by bath application of CB1 receptor agonist CP 55,940 (2 µM) (Valiullina et al., 2017). To determine evoked GABA release in the presence of glucose or lactate, we measured the effect of the low-affinity competitive $GABA_A$ receptor antagonist TPMPA (Jones et al., 2001), on the amplitudes of IPSCs evoked by electrical stimulation (10 Hz and 40 Hz).

Patch electrodes were pulled from hard borosilicate capillary glass (Flaming/Brown Micropipette Puller, Sutter Instruments, Novato, CA, USA). Electrodes for the current-clamp experiments were filled with a solution, which consisted of (in mM): 110 K-gluconate, 30 KCl, 10 HEPES, 8 NaCl, 4 MgATP, 0.3 MgGTP, 10 phosphocreatine, (pH 7.3 with KOH). The high intracellular Cl-concentration improves the signal-to-noise ratio and causes depolarizing IPSPs recorded at resting membrane potential (~ -70 mV). For recordings of EPSCs, postsynaptic interneurons were patched with solution containing (in mM): 110 Cs-gluconate, 30 CsCl, 10 HEPES, 8 NaCl, 4 MgATP, 0.3 MgGTP, 10 phosphocreatine (pH 7.3 with CsOH). The high intracellular Cs-concentration permitted to also record dendritic EPSCs with improved space-clamp conditions. Recordings were made using an EPC-10 amplifier (HEKA Elektronik GmbH, Lambrecht (Pfalz), Germany). Stimulus delivery and data acquisition were performed using Patchmaster software (HEKA Elektronik).

Optogenetics

Optogenetical methods were used to evoke theta-gamma oscillations in slice cultures. At DIV 4 slice cultures were infected with an adeno-associated viral vector (AAV-CaMKIIα-hChR2(H134R)-mCherry, UNC Gene Therapy Center Vector Core, Chapel Hill, NC, USA). 1 µl virus was carefully applied onto the CA3 region of each slice. Slice cultures were then maintained in the incubator for at least 3 weeks for expression of humanized channelrhodopsin2 (hChr2) in pyramidal cells under the control of the CaMKIIα-promotor. For recordings, the intact Biopore™ membrane carrying slice cultures was inserted into the interface type recording chamber (see above). To evoke theta-gamma oscillations, slice cultures where excited with blue light (470 nm) from an LED. The light intensity of the LED was modulated with a sinusoidal intensity profile, with a frequency of 5 Hz (theta). Blue light was delivered to the entire slice culture, whereas LFPs were recorded in stratum pyramidale of the CA3 region.

Tissue oxygen concentration and CMRO$_2$

The oxygen concentration was measured at different depths in stratum pyramidale of the CA3 region by using oxygen sensor microelectrodes (O$_2$-sensor), i.e., standard OX-10 (Unisense A/S, Aarhus, Denmark). This modified polarographic Clark electrode consists of a glass-insulated Ag/AgCl reference anode and a guard cathode with the advantages of low sensitivity to motion artifact, minimal interaction with tissue, and low O$_2$ consumption. The standard OX-10 has a tip diameter of 8 to 12 µm and a spatial resolution of the outside tip diameter. The O$_2$-sensor was connected to a 4-channel microsensor multimeter (Unisense A/S) and polarized with -0.8 V overnight. For recordings, the O$_2$-sensor was fixed in a mechanical micromanipulator at an angle of 60° and moved forward in steps of 23 µm (corresponding to a vertical depth of ~20 µm per step). Before and after each experiment, O$_2$-sensors were individually calibrated using a two point calibration with aCSF saturated with 0% O$_2$ + 100% N$_2$ and 95% O$_2$ + 5% CO$_2$, respectively (Kann et al., 2011; Schneider et al., 2019). Changes in voltage were digitized on-line at 10 kHz and data were stored on a computer disk with Sensor Trace Basic (data rate: 10 samples/s, Unisense A/S) for offline analysis. The oxygen concentration at a given depth in the slice can be described by a reaction diffusion model (Hall et al., 2012; Huchzermeyer et al., 2013; Schneider et al., 2019) given by the following differential equation:

$$D \cdot \frac{\mathrm{d}^2 cO_2}{\mathrm{d}x^2} = A \cdot \frac{cO_2}{cO_2 + K_m}$$

cO_2: oxygen concentration [µM] at different depths in the slice
x: depth in the slice
D: diffusion constant $1.6 \cdot 10^{-3} \mathrm{\mu m}/s$
K_m: enzymatic properties of the respiratory chain 4.7 µM
A: oxygen consumption rate (CMRO$_2$) [µM/s]

The CMRO$_2$ was estimated by fitting the solution of this differential equation to the experimentally measured oxygen depth profile, where CMRO$_2$ was the fitting parameter. The differential equation was solved with MATLAB (MathWorks, Natick, MA, USA) using the bvp4c-function. Two boundary conditions were used: 1) the oxygen concentration was set to the measured value at the slice surface. 2) The gradient of the oxygen concentration was set to 0 at the minimal oxygen concentration measured. The model describes the oxygen dynamics, which depend on diffusive oxygen transport and the metabolic oxygen consumption rate within a slice. For each depth profile derived from the experiments, we determined the maximal CMRO$_2$ by minimizing the square distance (R^2-value) between values measured by the O$_2$-sensor and the simulation of the reaction diffusion model. The model takes into account the physiological capillary pO$_2$ and the saturation of the respiratory chain and was validated by oxygen depth profiles from dead hippocampal slices (Kann et al., 2011). For more detailed information on the mathematical model, see Schneider et al., (2019). Please note that the CMRO$_2$ for a given brain tissue is also reported as µmol/g/min (Erecińska and Silver, 2001; Okada and Lipton, 2007; Zhu et al., 2007). To convert these values in mM/min (i.e. mmol/l/min) as provided in the present study, we assumed that the density of gray matter is roughly about 1.05 g/ml (Kasischke et al., 2011). Thus, the CMRO$_2$ of about 1.8 µmol/g/min measured in anaesthetized rats at 37°C corresponds to approximately 1.9 mM/min (Zhu et al., 2007; Engl et al., 2017).

Data analysis

Offline analysis was performed in MATLAB (MathWorks) using custom written routines. For analysis of pharmacologically induced gamma oscillations, data segments of 5 min were subdivided into segments of 30 s, band-pass filtered (FFT filter, pass-band frequency: 5 - 200 Hz) and processed with Welch's algorithm and a fast Fourier transformation (FFT size: 8192). The resulting power spectral density (PSD) plots had a resolution of 1.2207 Hz. Gamma oscillations were analyzed for various parameters, i.e., peak power spectral density (Power), peak frequency (f), area under the curve (AuC), full width at half maximum (FWHM) and TAU (Kann et al., 2011; Schneider et al., 2019). Power, FWHM and TAU primarily reflect number of activated synapses, synchronization and inner coherence, respectively. AuC reflects power and frequency width. Similarity and lag of gamma oscillations between CA1 and CA3 were calculated from cross correlation's 1st peak amplitude and shift, respectively. Medians of subdivisions were calculated and used for further statistical analysis. Power and peak frequency of optogenetically induced gamma oscillations were calculated from wavelet transformations of data recorded during light stimulation. For statistical evaluation, we compared means from data segments of 1 min at an early (1.5 - 2.5 min) and late stage (4 - 5 min) of the experimental condition.

To analyze SPW-Rs, signals were first separated into their slow (sharp wave) and fast components (ripples). The slow component was obtained by low-pass filtering (FFT filter, cut frequency: 45 Hz) and used for event detection and calculation of amplitude and duration. The ripple component was isolated by a band-pass filter (FFT filter, pass-band frequency: 120 - 400 Hz). Ripples were counted only when subsequent ripples crossed a threshold of 3 times the standard deviation (SD) of the band-pass filtered signal. For statistical evaluation, we compared 3 segments of 5 min.

Analysis of patch-clamp recordings was performed using IGOR PRO (WaveMetrics Inc., Portland, OR, USA) and Sigmaplot (Systat Software Inc., San Jose, CA, USA) (Rozov et al., 2001; Watanabe et al., 2005; Valiullina et al., 2017).

Data are summarized by their median ± the interquartile range (IQR = 75% percentile - 25% percentile) if not stated otherwise. The error bars indicate the minimal and the maximal values. Number of slices (n) from rats or preparations (N) are given in the legends.

Statistical evaluation

Statistical evaluation was done in Prism (GraphPad Software Inc., La Jolla, CA, USA). If data were normally distributed (Shapiro-Wilk), statistical evaluation was performed by a one-way analysis of variance (ANOVA) with Holm-Šídák's correction for multiple comparisons to identify significant differences between more than two conditions. If data were not normally distributed (Shapiro-Wilk), non-parametric tests (Kruskal–Wallis as well as Friedman) were used and followed by Dunn's multiple comparisons or Tukey's pairwise comparisons test. Comparison of two groups was done by t-tests (two-tailed) for normally distributed data, otherwise Wilcoxon's (paired) or Mann-Whitney's (unpaired) tests were used. p-Values less than 0.05 were considered to indicate a significant difference between groups (indicated by asterisks).

Supplemental References

Bahr, B.A., Kessler, M., Rivera, S., Vanderklish, P.W., Hall, R.A., Mutneja, M.S., Gall, C., and Hoffman, K.B. (1995). Stable maintenance of glutamate receptors and other synaptic components in long-term hippocampal slices. Hippocampus *5*, 425-439. DOI:10.1002/hipo.450050505.

Bischofberger, J., Engel, D., Li, L., Geiger, J.R.P., and Jonas, P. (2006). Patch-clamp recording from mossy fiber terminals in hippocampal slices. Nat. Protoc. *1*, 2075-2081. DOI:10.1038/nprot.2006.312.

De Simoni, A., Griesinger, C.B., and Edwards, F.A. (2003). Development of rat CA1 neurones in acute versus organotypic slices: role of experience in synaptic morphology and activity. J. Physiol. *550*, 135-147. DOI:10.1113/jphysiol.2003.039099.

Engl, E., Jolivet, R., Hall, C.N., and Attwell, D. (2017). Non-signalling energy use in the developing rat brain. J. Cereb. Blood Flow Metab. *37*, 951-966. DOI:10.1177/0271678X16648710.

Erecińska, M., and Silver, I.A. (2001). Tissue oxygen tension and brain sensitivity to hypoxia. Respir. Physiol. *128*, 263-276. DOI:10.1016/S0034-5687(01)00306-1.

Fleidervish, I.A., Friedman, A., and Gutnick, M.J. (1996). Slow inactivation of Na^+ current and slow cumulative spike adaptation in mouse and guinea-pig neocortical neurones in slices. J. Physiol. *493 (Pt 1)*, 83-97. DOI:10.1113/jphysiol.1996.sp021366.

Haas, H.L., Schaerer, B., and Vosmansky, M. (1979). A simple perfusion chamber for the study of nervous tissue slices in vitro. J. Neurosci. Methods *1*, 323-325. DOI:10.1016/0165-0270(79)90021-9.

Hájos, N., and Mody, I. (2009). Establishing a physiological environment for visualized *in vitro* brain slice recordings by increasing oxygen supply and modifying aCSF content. J. Neurosci. Methods *183*, 107-113. DOI:10.1016/j.jneumeth.2009.06.005.

Hu, H., and Jonas, P. (2014). A supercritical density of Na^+ channels ensures fast signaling in GABAergic interneuron axons. Nat. Neurosci. *17*, 686-693. DOI:10.1038/nn.3678.

Ivanov, A.I., and Zilberter, Y. (2011). Critical state of energy metabolism in brain slices: the principal role of oxygen delivery and energy substrates in shaping neuronal activity. Front. Neuroenergetics *3*, 9. DOI:10.3389/fnene.2011.00009.

Kann, O. (2012). The energy demand of fast neuronal network oscillations: insights from brain slice preparations. Front. Pharmacol. *2*, 90. DOI:10.3389/fphar.2011.00090.

Kasischke, K.A., Lambert, E.M., Panepento, B., Sun, A., Gelbard, H.A., Burgess, R.W., Foster, T.H., and Nedergaard, M. (2011). Two-photon NADH imaging exposes boundaries of oxygen diffusion in cortical vascular supply regions. J. Cereb. Blood Flow Metab. *31*, 68-81. DOI:10.1038/jcbfm.2010.158.

McIlwain, H. (1951). Metabolic response *in vitro* to electrical stimulation of sections of mammalian brain. Biochem. J. *49*, 382-393. DOI:10.1042/bj0490382.

Okada, Y., and Lipton, P. (2007). 1.2 Glucose, Oxidative Energy Metabolism, and Neural Function in Brain Slices—Glycolysis Plays a Key Role in Neural Activity. In Handbook of Neurochemistry and Molecular Neurobiology. Brain Energetics. Integration of Molecular and Cellular Processes, A. Lajtha, G.A. Dienel and G.E. Gibson, eds. (Boston, MA: Springer-Verlag), pp. 17–39. DOI:10.1007/978-0-387-30411-3_2.

Partin, K.M., Patneau, D.K., and Mayer, M.L. (1994). Cyclothiazide differentially modulates desensitization of α-amino-3-hydroxy-5-methyl-4-isoxazolepropionic acid receptor splice variants. Mol. Pharmacol. *46*, 129-138.

Schmitz, D., Empson, R.M., and Heinemann, U. (1995). Serotonin reduces inhibition via 5-HT_{1A} receptors in area CA1 of rat hippocampal slices *in vitro*. J. Neurosci. *15*, 7217-7225. DOI:10.1523/JNEUROSCI.15-11-07217.1995.

Zhu, X.-H., Zhang, Y., Zhang, N., Ugurbil, K., and Chen, W. (2007). Noninvasive and three-dimensional imaging of $CMRO_2$ in rats at 9.4 T: reproducibility test and normothermia/hypothermia comparison study. J. Cereb. Blood Flow Metab. *27*, 1225-1234. DOI:10.1038/sj.jcbfm.9600421.
